# Poverty and health care demand in Kenya

**DOI:** 10.1186/s12913-014-0560-y

**Published:** 2014-11-22

**Authors:** Japheth Osotsi Awiti

**Affiliations:** School of Economics, University of Nairobi, University Way, Nairobi, Kenya

**Keywords:** Poverty, Health care demand, Kenya

## Abstract

**Background:**

There is a wide range of actions an individual could take when sick or injured such as self–care, consulting a traditional healer, or seeking treatment from a private or public health care facility. The specific action taken is influenced by individual characteristics, provider characteristics, societal factors, and geographical factors. A key individual characteristic is the ability to afford the required health care. The study examines the effect of poverty on an individual’s choice of a health care provider in the event of sickness or injury in Kenya.

**Methods:**

Using data from the Kenya Integrated Household and Budget Survey carried out between 2005 and 2006, we estimate a multinomial probit model that links an individual’s poverty status to the individual’s health care provider choice. The choices are classified as none, non-modern, and modern. The model is estimated for four age groups: infants, children aged 1 to 5 years, children aged 6 to 14 years, and adults. We control for the potential endogeneity of poverty status.

**Results:**

Our results indicate that for all age groups, the predictors of poverty include large household sizes and longer distances to the nearest health facility. We further find that poverty reduces the probability of visiting a modern health care provider amongst all age groups.

**Conclusions:**

Poverty has a negative effect on the individual’s demand for modern health care services, holding other factors constant. To encourage the use of modern health care facilities, therefore, requires the pursuit of poverty–reduction strategies. Some of the ways this could be done include lowering the household sizes and reducing the average distance to modern health care facilities.

**Electronic supplementary material:**

The online version of this article (doi:10.1186/s12913-014-0560-y) contains supplementary material, which is available to authorized users.

## Background

Table 1 below gives some key health indicators for Kenya, Rwanda, and the averages for Europe.

**Table 1:** **Key health indicators**

**Indicator**	**Kenya**	**Rwanda**	**Europe**
Life expectancy at birth (years), 2011	60	60	76
Still birth rate (per 1000 total births), 2009	22	23	6
Neonatal mortality rate (per 1000 live births), 2011	27	21	6
Infant mortality rate (per 1000 live births), 2011	48	38	11
Under–five mortality rate (per 1000 live births), 2011	73	54	13
Maternal mortality ratio (per 100,000 live births), 2010	360	340	20

Source: [1].

The table clearly shows that Rwanda performs better than Kenya on all the indicators except life expectancy at birth, and still birth rate. The table further shows that Kenya trails Europe on all the indicators. The fact that Rwanda is ahead of Kenya on nearly all the indicators clearly indicates that more needs to be done by Kenya to improve these indicators. Studies that, therefore, provide a way forward on how these indicators can be improved are welcome. This study attempts to do this, although indirectly.

Since health is an important component of human capital, good health can substantially increase the capabilities of individuals to perform various activities, including income–generating ones [2,3]. As a result, individuals demand good health [4].

Health, at the individual level, is mainly influenced by a variety of factors such as unobservable biological determinants, lifestyle choices (also referred to as health–related behaviours), non–medical purchased inputs, purchased medical inputs (health care), and various socio–economic factors [4-8]. This study is concerned with one of these determinants: health care.

When sick or injured, there is a wide range of actions that an individual can take as far as health care is concerned. These actions include self–care, consulting traditional healers, or seeking health care from various private and public health care facilities [9-15]. The specific action taken by the individual is influenced by various factors such as individual/household characteristics, the characteristics of the various health care providers (particularly price of obtaining care and quality of care), various societal factors, and geographical factors (such as seasonality) [16-19].

The key societal factors include technology (the principles and techniques that influence available care) and norms (the modes through which a society induces and ensures normal compliance by members) [16]. Price of obtaining care includes the direct price paid for the treatment and indirect prices such as travel costs, opportunity (time) costs and any informal payments made at a health care facility to facilitate treatment [18]. Quality of care is broadly defined to include structural, process, and outcome dimensions [19]. Geographical factors such as seasonality have the potential to substantially raise the opportunity cost of time spent seeking treatment in the rural areas, especially during the rainy season [17].

The individual characteristics include the individual’s predisposition to seek health care when in ill–health, the individual’s ability to secure the required health care, and the level of the illness the individual suffers from [16,20].

One of the key determinants of the individual’s ability to secure the required health care is the individual’s material possessions in the form of income and/or assets [20]. The effect of income on the demand for health care has been studied in the literature in various ways such as by investigating the effect of income on health seeking behaviour, by investigating the effect of income on health expenditures or by investigating the effect of poverty on health care demand [21,22]. The studies in the literature that try to investigate the effect of poverty on health care demand have, however, been mainly done at the aggregate level (that is, levels higher than the household level).

In this study, we examine the effect of the poverty status of an individual on the choice of a health care provider by the individual when sick or injured in Kenya. The general objective of the study is to, therefore, establish how poverty influences health care demand through its effects on health care provider choice at the individual level. Specifically, we classify individuals in each household as either poor or otherwise depending on the poverty status of the household to which they belong. We next classify the range of health care providers the individual reports to have consulted when ill as either none, non–modern, or modern. We then link the individual’s poverty status to the type of health care provider the individual reports to have consulted when sick or injured. The results of our analysis are then used to draw some policy implications.

Our study makes several contributions to the literature. First, unlike previous studies, we examine the effect of poverty status on health care provider choice at the individual level. Second, we provide evidence of the effect of poverty status on the demand for health care from Kenya, a developing country. Third, in estimating the effect of poverty status on health care provider choice, we explicitly take into account the endogeneity of poverty status in the health care provider choice equation.

## Methods

This section explains the methods of the study. The section discusses the theoretical framework for the study, the conceptual framework, the estimation issues, the identification strategy, the empirical model and the data.

### Theoretical framework

**Figure 1:**
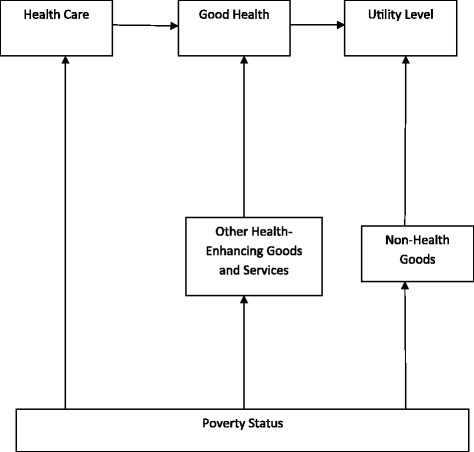
**A Theoretical Framework for Analyzing the Effect of Poverty Status on Health Care Demand.** The figure shows how the poverty status of the individual constrains the individual’s utility maximization objective.

We can develop the theoretical framework shown in Figure 1 below based on [20].

The starting point in the figure is that individuals within a household, are assumed to derive utility from the consumption of non–health goods (such as clothing) and good health. To produce good health, however, the individuals have to consume health care and other health–enhancing goods. The poverty status of the household, however, limits the kind of health care, other health–enhancing goods, and the non–health goods that the household members can consume. The individuals are assumed to choose their consumption levels of health care, other health–enhancing goods, and non–health goods in such a way that their overall utility is maximized.

### Conceptual framework

**Figure 2:**
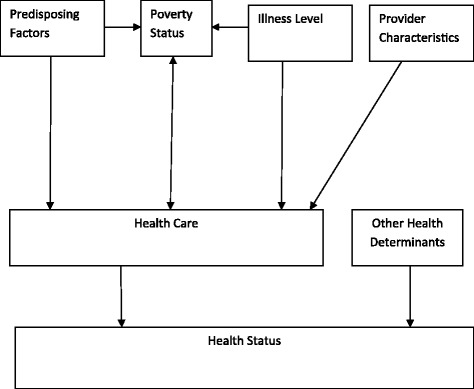
**A Conceptual Framework for Analyzing the Effect of Poverty Status on Health Care Demand.** The figure shows how the various factors are related to the demand for health care.

We present in Figure 2, shown below, a conceptual framework for analyzing the effect of poverty status on the health care demand by an individual. The figure is constructed based on [16,19].

According to the figure, health care and other factors determine an individual’s health status. The individual, therefore, demands health care as an input into his/her health production process. The demand for health care is influenced by predisposing factors such as age, sex, marital status, education, religion, household size, education, etc; enabling factors such as the poverty status of the individual/household; illness level; and provider characteristics such as price of care and quality of care. The poverty status is, in turn, influenced by the predisposing factors, the demand for health care (mainly due to catastrophic health care expenditures) [23-25], and the illness level. The arrows in the figure do not necessarily imply causality but do indicate the direction of influence.

### Estimation issues

Whenever we estimate a model based on survey data we need to, in general, worry about three main issues that have the potential to bias our parameter estimates: sample selection bias, endogeneity of some of the covariates, and unobserved heterogeneity.

In our dataset, individuals were asked whether they were sick or injured in the four weeks period immediately preceding the survey. Those who answered in the affirmative were then asked whether or not they consulted a health provider. In this case, sample selection bias will arise if the unobservable factors influencing the decision to report whether or not one consulted a health provider are correlated with the unobservable factors influencing actually consulting a health provider [26]. This problem does not, however, exist in our case since 99.95% of those who reported being sick or injured in the four weeks prior to the survey also reported whether or not they had consulted a health provider. It is only 0.05% we are not sure of, which could actually be a case of data–entry errors.

An individual’s poverty status is potentially endogenous in our model due to the potential reverse causality between the poverty status and the health care provider choice [27,28]. There is, therefore, the risk of inconsistency of our estimated coefficients and the further risk of inability to infer causality between the poverty status and health care provider choice [28]. We can, however, use the method of Two–Stage–Residual–Inclusion (2SRI) to consistently estimate our model [29]. This technique involves two steps. In the first step, we estimate a poverty status equation and obtain the generalized residuals using the methods discussed in [30]. In the second step, we estimate the health care provider choice equation where both the poverty status variable and its generalized residuals are included in the set of explanatory variables. Poverty status will be endogenous in the health care provider choice equation if the coefficient of the generalized residuals is statistically different from zero [31].

Unobserved heterogeneity will occur in our model if there is a non–linear interaction between unobservable factors and poverty status that cause the effect of poverty status on health care provider choice to differ amongst population subjects [32]. We control for unobserved heterogeneity using the control function approach [33]. This approach generally involves including in the health care provider choice equation interactions between the generalized residuals from the poverty status equation and the poverty status variable. A coefficient of the resulting interaction term that is statistically significantly different from zero is indicative of the presence of unobserved heterogeneity in the health care provider choice equation.

### Model identification

Since it is only the poverty status variable in our model that is assumed to be endogenous, identification of our model requires one exclusion restriction. The exclusion restriction, also referred to as the instrumental variable, should not be correlated with the stochastic error term in the health care provider choice equation (that is, it should be valid), should be correlated with the poverty status in the health care provider choice equation (that is, it should be relevant), and should be excluded from the health care provider choice equation [34].

We use the proportion of children that are severely underweight in each district as an instrumental variable for poverty status. This choice is motivated by two main reasons. First, we do not expect the proportion of children who are severely underweight in a district to affect the demand for health care at the individual/household level. Second, as shown in the literature (see, for example, [35]), there is a high correlation between childhood undernutrition and poverty. We, therefore, expect that households found in districts with higher proportions of severely underweight children to have a higher probability of being poor and vice–versa.

### Empirical model

Based on the conceptual framework in Figure 2, we can formulate the following health care demand function for an individual in a particular household who reports having been sick or injured in the four weeks immediately preceding the survey

(1) $$ \text{Health Care Provider}=F\left(\text{PS}, \text{PF}, \text{IL}, \text{PC}; u\right)  $$

where PS is poverty status, PF are predisposing factors, IL is illness level, PC is price of care, and *u* is the error term capturing unobservable influences on health care provider choice.

Specifically, the Health Care Provider (HCP) variable is defined as follows:

(2) $$ HCP=\left\{ \begin{array}{l} 1\,\,\, \text{if no provider is consulted} \\ 2 \,\,\, \text{if a non-modern provider is consulted} \\ 3 \,\,\, \text{if a modern provider is consulted} \end{array} \right.  $$

while the poverty status variable is defined as

(3) $$ PS=\left\{ \begin{array}{l} 1 \hspace*{0.2in} \text{if household is poor} \\ 0 \hspace*{0.2in} \text{otherwise} \end{array} \right.  $$

As explained earlier, the first step of the 2SRI technique requires that we estimate the poverty status equation and obtain generalized residuals from it which are then used as an additional explanatory variable in the second step. The poverty status equation is given by

(4) $$ \text{PS}=F\left(\text{PF}, \text{IL}, \text{PC}, \text{PSU}; v\right)  $$

where PSU = Proportion of Severely Underweight Children in District and *v* is the error term capturing unobservable influences on poverty status.

To control for potential endogeneity of poverty status in Equation (1), the equation is re–estimated with the generalized residuals included as an additional independent variable. Controlling for potential unobserved heterogeneity involves further including in the equation interactions between poverty status and the generalized residuals.

Since the poverty status variable is binary, we can either use the probit model or the logit model to estimate Equation (4) [36]. We choose the probit model since according to the literature, it does not matter in general whether we use the probit or logit model as the coefficients obtained using one model can be transformed into those obtained using the other model [36,37].

The HCP variable, on the other hand, is polychotomous. Two main models for estimating such a variable include the multinomial logit model and the multinomial probit model [36,38]. The multinomial logit model, however, has the disadvantage of imposing the assumption of the independence of irrelevant alternatives (IIA) on our choices, which may be untenable in practice [36]. To avoid the problems posed by the IIA assumption, therefore, we use the multinomial probit model to estimate the health care provider choice models [38].

### Data

We use data from the Kenya Integrated Household Budget Survey conducted in 2005/2006 [39]. The survey collected data on various socio–economic aspects from a national representative sample of over 10,000 households [39]. Data on poverty status of the households come from a report on poverty compiled using the survey data [40]. Data on the proportion of children who are severely underweight for each district is obtained from Table 6.1 of the survey report [39].

### Ethics

The data used in this study were not collected by the author directly but by the Kenya National Bureau of Statistics (KNBS), a public body mandated to collect data from the Kenyan population. Most of the ethical issues that would, therefore, arise from data collected by the researcher do not arise in this case. Chapter 2 of the report accompanying the data contains detailed information on the design of the survey and the actual process of data collection [39]. It can also be inferred from the survey questionnaire that participation in the survey was voluntary for the sampled households. The survey questionnaire that was used by the Kenya National Bureau of Statistics to collect the data used in this study has been provided as an Additional file 1.

## Results

This section presents the results of the study. We first show the variable definitions and then the descriptive statistics. The results of the estimations of our models, both from the first step and the second step, are also presented.

Our analytical sample consists of those individuals who reported being sick or injured in the four weeks immediately preceding the survey. The unit of analysis is the individual household member.

### Variable definitions

Table 2 shows the variable definitions for the variables in our models.

**Table 2:** **Variable definitions**

**Variable**	**Definition**
Health care provider	1 if individual did not consultany health care provider,
	2 if the individual consulted anon–modern health care provider,
	3 if the individual consulted amodern health care provider.
Poor	1 if household to which theindividual belongs is poor;
	0 otherwise.
Rural	1 if individual’s area of residenceis rural; 0 otherwise.
Male	1 if individual is male; 0 otherwise.
Infant age	Age of infant in months.
Household size	Number of members in household.
Fever/Malaria	1 if individual’s illness is fever ormalaria; 0 otherwise.
Flu	1 if individual’s illness is flu;0 otherwise.
Headache	1 if individual’s illness is headache;0 otherwise.
Other illness	1 if individual suffers from illnessesother than, fever/malaria, flu orheadache; 0 otherwise.
Severe underweight	Proportion of children that areseverely underweight in districtwhere the household resides.
Age	Individual’s age in years.
Age squared	The square of individual’s age.
Catholic	1 if individual is catholic; 0 otherwise.
Protestant	1 if individual is protestant;0 otherwise.
Muslim	1 if individual is muslim; 0 otherwise.
Other religion	1 if individual’s religion is neithercatholic, protestant nor muslim;0 otherwise.
No education	1 if individual has no formaleducation; 0 otherwise.
No education—household head	1 if household head has no formaleducation; 0 otherwise.
Married	1 if individual is married; 0 otherwise.
Married—household head	1 if household head is married;0 otherwise.
Distance to nearest health facility	Average distance to nearest healthfacility in kilometres.
	Computed at the district level.
Poor residuals	Generalized residuals for the povertystatus model.
Poor interacted with residuals	Poverty status interacted withgeneralized residuals from thepoverty status model.

### Descriptive statistics

The descriptive statistics are shown in Table 3.

**Table 3:** **Descriptive statistics, the entire sample**

**Variable**	**Number of**	**Mean**	**Standard**	**Minimum**	**Maximum**
	**observations**		**deviation**		
Health care provider	17,252	2.3254	0.9048	1	3
Poor	17,285	0.4747	0.4994	0	1
Rural	17,285	0.7203	0.4489	0	1
Male	17,285	0.4478	0.4973	0	1
Infant age	733	6.5634	3.0019	1	11
Household size	17,285	6.1345	2.9466	1	29
Fever/Malaria	17,285	0.4261	0.4945	0	1
Flu	17,285	0.1381	0.3450	0	1
Headache	17,285	0.0583	0.2343	0	1
Other illness	17,285	0.3775	0.4848	0	1
Severe underweight	17,285	0.0468	0.0276	0	0.15
Age	16,552	25.0521	21.2424	1	97
Age squared	16,552	1078.822	1557.066	1	9409
Catholic	17,285	0.1795	0.3838	0	1
Protestant	17,285	0.3130	0.4637	0	1
Muslim	17,285	0.0616	0.2404	0	1
Other religion	17,285	0.4459	0.4971	0	1
No education	17,285	0.4194	0.4935	0	1
No education—household head	17,285	0.2730	0.4455	0	1
Married	17,285	0.3175	0.4655	0	1
Married—household head	17,285	0.7743	0.4180	0	1
Distance to nearest health facility	17,285	8.4843	9.8601	2.6	58

From the table, we can observe that about 47.47% of the individuals in our sample come from poor households. We can further observe from the table that the number of members in the household ranges from 1 to 29. The table also indicates that about 42.61% of the individuals reporting being sick or injured had fever or malaria while the average age of infants in our sample is 6.5634 months. The table further shows that the average distance to the nearest health facility ranges from 2.6 kilometres to 58 kilometres.

Table 4 shows the type of health care provider visited by those who were sick or injured in the last four weeks preceding the survey.

**Table 4:** **Type of facility visited, the entire sample**

**Facility type**	**Percentage**	**Health care provider type**		
	**(%)**	**None**	**Non–modern**	**Modern**
Referral hospital	1.35	0	0	1
District/Provincial hospital	11.91	0	0	1
Public dispensary	13.92	0	0	1
Public health center	9.45	0	0	1
Private dispensary/ hospital	7.36	0	0	1
Private clinic	6.76	0	0	1
Traditional healer	0.15	0	1	0
Missionary hospital/ dispensary	2.57	0	0	1
Pharmacy/chemist	9.16	0	0	1
Kiosk	6.05	0	1	0
Faith healer	0.09	0	1	0
Herbalist	0.86	0	1	0
Other	0.41	0	1	0
None	30	1	0	0
**Total**	100	30	7.56	**62.48**

The various health care providers have also been classified in the table as either none, non–modern or modern. We consider a health care provider to be modern if one can obtain formal health care goods and services from the provider [16]. One cannot obtain such goods and services from non–modern health care providers.

In the table, we can observe that the health care providers visited range from referral hospitals to not visiting any health care provider. Health care providers classified as modern include referral hospitals, district/provincial hospitals, public dispensaries, public health centers, private dispensaries/hospitals, private clinics, missionary hospitals/dispensaries, and pharmacies/chemists. The non–modern health care providers include traditional healers, kiosks, faith healers, herbalists, and facilities classified as “other”.

The table shows that 30% of individuals in our sample who reported being sick or injured in the four weeks preceding the survey did not consult any health care provider, 7.56% consulted non–modern health care providers, while 62.48% consulted modern health care providers. A further look at the table shows, for example, that 1.35% visited a referral hospital, 0.15% visited a traditional healer, while 13.92% visited a public dispensary.

### Poverty status models

We first estimate the poverty status models. We have decided to subdivide our sample into four categories: infants (aged under 1 year), children (aged 1 to 5 years), children (aged 6 to 14 years), and adults (aged 15 years and above). The reasons for the subdivision of the sample into subsamples based on age groups are twofold. First, for some of the age groups some of the variables are measured in different units. For example, age is measured in months for infants but in years for the other age groups. Second, some of the variables do not make sense for certain age groups. For example, marital status and level of education do not make sense for infants, children aged 1 to 5 years, and children aged 6 to 14 years. In such models, therefore, we include the education level of the household head and the marital status of the household head, instead.

For each age group, we estimate a probit model. We report average marginal effects. The average marginal effects are obtained as follows: we first compute the marginal effect for the respective covariate for each observation in the subsample, and then find the arithmetic mean of these marginal effects for all the observations [36]. The results are shown in Table 5 below.

**Table 5:** **Average marginal effects for the poverty status models, robust** ***Z*** **statistic in parentheses**

	**Dependent variable = poor**			
**Variable**	**Infants**	**Children**	**Children**	**Adults**
	**(Age** ***<*** ** 1)**	**(Age 1–5)**	**(Age 6–14)**	**(Age** ***≥*** ** 15)**
Rural	-0.0002	0.013	-0.001	0.009
	(-0.01)	(0.71)	(-0.08)	(0.78)
Male	-0.048	-0.015	0.041	-0.014
	(-1.39)	(-0.93)	(2.63)	(-1.50)
Infant age	0.006			
	(1.03)			
Household size	0.03	0.046	0.042	0.044
	(4.04)	(12.77)	(13.00)	(27.05)
Fever/Malaria	-0.036	-0.023	-0.061	-0.029
	(-0.93)	(-1.21)	(-3.40)	(-2.76)
Flu	0.051	-0.002	-0.021	-0.028
	(1.06)	(-0.08)	(-0.86)	(-1.73)
Headache	0.004	0.117	0.069	0.059
	(0.01)	(1.49)	(2.08)	(3.19)
Distance to nearest health facility	0.007	0.005	0.008	0.007
	(3.49)	(5.51)	(7.92)	(12.47)
No education— household head	0.113	0.126	0.13	
	(2.96)	(6.86)	(7.48)	
Married—household head	-0.081	-0.024	-0.078	
	(-1.56)	(-1.08)	(-3.95)	
Age		-0.007	-0.023	0.002
		(-1.17)	(-0.84)	(7.58)
Age squared			0.001	
			(1.11)	
Catholic			-0.049	-0.022
			(-1.60)	(-1.50)
Protestant			-0.045	-0.046
			(-1.73)	(-3.50)
Muslim			0.008	0.029
			(0.19)	(1.46)
No education				0.121
				(12.17)
Married				-0.032
				(-3.26)
Severe underweight	2.869	1.838	2.47	1.85
	(3.74)	(5.67)	(8.05)	(10.06)
Number of observations	733	3,315	3,530	9,707

The results in the table indicate that for all age–groups, the predictors of poverty include large household sizes and longer distances to the nearest health facility. In particular, an increase in the household size by one member increases the probability of being poor among infants by 0.03, among children aged 1 to 5 years by 0.046, among children aged 6 to 14 years by 0.042, and among adults by 0.044, holding other factors constant. Further, an increase in the average distance to the nearest health facility by 1 kilometre increases the probability of being poor by 0.007 among infants, by 0.005 among children aged 1 to 5 years, by 0.008 among children aged 6 to 14 years, and by 0.007 among adults, holding all the other factors constant.

Among the children, poverty is higher for those children who live in households where the household head has no formal education compared to those who live in households where the household head has formal education, holding other factors constant. In particular for children aged 1 to 5 years, poverty is higher among households where the household head has no formal education as compared to those where the household head has formal education by 0.126, holding other factors constant. Among the children aged 6 to 14 years, poverty is higher for those households where the household head has no formal education compared to those households where the household head has formal education by 0.13, holding other factors constant.

Among the adults, poverty is higher amongst those suffering from headaches (as compared to other illnesses), the older ones, and those without formal education. For example, the results show that compared to adults with formal education, those without formal education have a higher probability of being poor by 0.121, holding other factors constant.

The results also show that among adults, those who are married are less likely to be poor compared to those who are not married, holding other factors constant. In particular, being married as opposed to not being married reduces the probability of being poor by 0.032, holding other factors constant.

### Health care provider choice models

We also estimate health care provider choice models for all the age groups. For each age group we estimate three models: a basic model, a model that controls for endogeneity of poverty status, and a model that controls for unobserved heterogeneity. The estimation results show that even though poverty is endogenous in health care provider choice equations, there is no unobserved heterogeneity in our models. The estimation results (average marginal effects) are shown in the Appendix. As such, the appropriate models are those that control for the endogeneity of poverty status. We show in Tables 6, 7, 8 and 9 the estimation results for the models that control for the endogeneity of poverty status. Each of the tables shows the average marginal effects for the various types of health care providers for the different age groups. Table 6 shows the results for infants, Table 7 provides the results for children aged 1 to 5 years, Table 8 shows the results for children aged 6 to 14 years, while Table 9 shows the results for adults.

**Table 6:** **Average marginal effects for the health care provider choice models for infants (Age < 1 year), robust** ***Z*** **statistic in parentheses**

	**Dependent variable = health care provider**		
**Variable**	**None**	**Non–modern**	**Modern**
Poor	0.375	0.074	-0.449
	(1.93)	(0.70)	(-2.10)
Rural	-0.027	0.029	-0.002
	(-0.79)	(1.59)	(-0.05)
Male	0.027	0.029	-0.057
	(0.87)	(1.79)	(-1.68)
Infant age	-0.007	-0.001	0.008
	(-1.29)	(-0.52)	(1.44)
Household size	-0.0002	-0.002	0.003
	(-0.03)	(-0.48)	(0.28)
Fever/Malaria	-0.003	-0.013	0.015
	(-0.08)	(-0.71)	(0.42)
Flu	0.081	0.013	-0.095
	(2.02)	(0.71)	(-2.20)
Headache	0.403	-0.564	0.161
	(1.47)	(-6.18)	(0.54)
Distance to nearest health facility	-0.002	-0.002	0.002
	(-0.17)	(-1.59)	(0.91)
No education— household head	-0.048	-0.003	0.051
	(-1.22)	(-0.17)	(1.22)
Married—household head	-0.029	0.018	0.011
	(-0.63)	(0.76)	(0.21)
Poor residuals	-0.203	-0.045	0.248
	(-1.67)	(-0.70)	(1.87)
Number of observations	729	729	729

**Table 7:** **Average marginal effects for the health care provider choice models for children (Age 1–5 years), robust** ***Z*** **statistic in parentheses**

	**Dependent variable = health care provider**		
**Variable**	**None**	**Non–modern**	**Modern**
Poor	0.401	0.184	-0.585
	(2.99)	(3.05)	(-4.21)
Rural	-0.008	0.040	-0.032
	(-0.43)	(3.94)	(-1.73)
Male	0.013	0.009	-0.023
	(0.86)	(1.27)	(-1.42)
Household size	-0.009	-0.008	0.017
	(-1.30)	(-2.55)	(2.37)
Fever/Malaria	-0.019	-0.0009	0.020
	(-1.04)	(-0.09)	(1.05)
Flu	0.112	0.036	-0.148
	(5.45)	(3.55)	(-6.89)
Headache	-0.021	0.030	-0.009
	(-0.30)	(1.08)	(-0.13)
Distance to nearest health facility	0.0005	-0.003	0.002
	(0.39)	(-4.21)	(1.68)
No education— household head	-0.004	-0.011	0.015
	(-0.18)	(-0.97)	(0.61)
Married—household head	-0.004	-0.009	0.013
	(-0.20)	(-0.90)	(0.60)
Age	0.019	0.006	-0.025
	(3.52)	(2.16)	(-4.39)
Poor residuals	-0.217	-0.094	0.312
	(-2.64)	(-2.52)	(3.64)
Number of observations	3,306	3,306	3,306

**Table 8:** **Average marginal effects for the health care provider choice models for children (Age 6–14 years), robust** ***Z*** **statistic in parentheses**

	**Dependent variable = health care provider**		
**Variable**	**None**	**Non–modern**	**Modern**
Poor	0.224	0.222	-0.447
	(2.10)	(3.69)	(-4.06)
Rural	0.010	0.070	-0.080
	(0.53)	(5.38)	(-4.19)
Male	-0.001	0.0008	0.0006
	(-0.09)	(0.08)	(0.04)
Household size	0.002	-0.011	0.009
	(0.33)	(-3.30)	(1.63)
Fever/Malaria	0.006	0.024	-0.030
	(0.32)	(2.02)	(-1.54)
Flu	0.160	0.061	-0.221
	(6.97)	(4.37)	(-9.21)
Headache	0.097	0.065	-0.163
	(3.02)	(3.82)	(-4.84)
Distance to nearest health facility	-0.0003	-0.005	0.005
	(-0.23)	(-5.17)	(3.62)
No education— household head	0.008	-0.018	0.010
	(0.37)	(-1.43)	(0.42)
Married—household head	-0.005	-0.005	0.010
	(-0.24)	(-0.38)	(0.45)
Age	0.062	0.003	-0.065
	(2.30)	(0.21)	(-2.34)
Age squared	-0.003	-0.0001	0.003
	(-2.31)	(-0.12)	(2.30)
Catholic	-0.013	-0.0001	0.013
	(-0.41)	9-0.01	(0.40)
Protestant	-0.012	0.006	0.006
	(-0.46)	(0.41)	(0.21)
Muslim	-0.053	-0.055	0.108
	(-1.27)	(-1.80)	(2.47)
Poor residuals	-0.088	-0.118	0.206
	(-1.33)	(-3.13)	(3.02)
Number of observations	3,523	3,523	3,523

**Table 9:** **Average marginal effects for the health care provider choice models for adults (Age≥ 15 years), robust** ***Z*** **statistic in parentheses**

	**Dependent variable = health care provider**		
**Variable**	**None**	**Non–modern**	**Modern**
Poor	0.271	0.156	-0.427
	(3.33)	(3.31)	(-5.02)
Rural	0.001	0.047	-0.048
	(0.13)	(6.61)	(-4.22)
Male	0.016	0.010	-0.026
	(1.68)	(1.73)	(-2.59)
Household size	-0.009	-0.008	0.017
	(-2.34)	(-3.37)	(4.10)
Fever/Malaria	0.004	0.024	-0.028
	(0.42)	(3.60)	(-2.56)
Flu	0.192	0.085	-0.277
	(12.37)	(9.60)	(-16.84)
Headache	0.071	0.068	-0.139
	(3.87)	(6.78)	(-7.18)
Distance to nearest health facility	0.001	-0.003	0.002
	(1.44)	(-5.71)	(1.87)
Age	0.0007	-0.0005	-0.0003
	(2.28)	(-2.47)	(-0.79)
Catholic	-0.104	0.017	0.087
	(-7.43)	(1.93)	(5.88)
Protestant	-0.081	0.014	0.067
	(-6.15)	(1.66)	(4.79)
Muslim	-0.077	-0.026	0.103
	(-3.90)	(-1.92)	(4.89)
No education	-0.009	-0.016	0.025
	(-0.63)	(-1.89)	(1.67)
Married	0.007	-0.001	-0.006
	(0.73)	(-0.17)	(-0.60)
Poor residuals	-0.114	-0.081	0.196
	(-2.30)	(-2.81)	(3.76)
Number of observations	9,694	9,694	9,694

The results in Table 6 show that among infants, poverty increases the probability of not visiting any health care provider when ill but reduces the probability of visiting a modern health care provider, holding other factors constant. Specifically, the results show that among infants, poverty increases the probability of not visiting any health care provider by 0.375 but reduces the probability of visiting a modern health care provider by 0.449, holding other factors constant.

From Table 7, we can observe that among children aged 1 to 5 years when other factors are held constant, poverty increases the probability of visiting non–modern providers and that of not visiting any provider, but reduces the probability of visiting a modern health care provider. In particular, for this age group, poverty increases the probability of not visiting any provider by 0.401, increases the probability of visiting a non–modern health care provider by 0.184, but reduces the probability of visiting a modern health care provider by 0.585, holding other factors constant.

Table 8 also shows that holding other factors constant, for children aged 6 to 14 years, poverty increases the probability of not visiting any health care provider by 0.224, it increases the probability of visiting a non–modern health care provider by 0.222, but reduces the probability of visiting a modern health care provider by 0.447. The results in the table also show that living in the rural areas as opposed to living in urban areas increases the probability of visiting a non–modern health care provider by 0.070 but decreases the probability of visiting a modern health care provider by 0.080, holding all other factors constant. The table also shows that an increase in the average distance to the nearest health facility by one kilometre reduces the probability of visiting a non–modern health care provider by 0.005 but increases the probability of visiting a modern health care provider by 0.005, holding other factors constant.

Table 9 results show that among adults, poverty increases the probability of not visiting any provider by 0.271, it increases the probability of visiting a non–modern health care provider by 0.156, but it reduces the probability of visiting a modern health care provider by 0.427, holding all the other factors constant. The results in the table also show that when other factors are held constant, adults living in rural areas compared to those living in urban areas have a higher probability of visiting a non–modern health care provider by 0.047 but a lower probability of visiting a modern health care provider by 0.048. We can also see from the table that adult males are less likely to visit modern health care providers compared to adult females by 0.026, holding other factors constant. The results in the table further show that among adults, an increase in the average distance to the nearest health facility by one kilometre reduces the probability of visiting a non–modern health care provider by 0.003 but increases the probability of visiting a modern health care provider by 0.002, holding other factors constant.

## Discussion

In this section we discuss our findings.

### Poverty status models

The association of large household sizes with increased probability of being poor may be due to the fact that larger households have larger demands in terms of the amounts of resources needed to satisfy the household’s basic food and non–food needs. This association is confirmed by other studies in the literature [41-44].

The association of longer distances to health facilities with poverty may be because health facilities that are farther away from where people live are less accessible to the majority of the people and this makes it difficult for people to seek modern health care when ill [45], decreasing their chances of engaging in income–generating activities. The finding is supported by other studies from the literature [46-48].

Not having formal education may be a predictor of poverty due to the fact that education opens up a range of income–generating opportunities, such as employment in the formal sector, which may not be available to those without formal education. The literature actually shows that the higher one’s education level is, the higher the private returns to education [49]. The association of lack of formal education and poverty is supported by studies in the literature [41,43,44].

The negative correlation between being married and being poor could be because marriage may increase the resources available to the household by, for example, having a spouse who earns a higher income. Studies in the literature support this correlation [43,44].

### Health care provider choice models

A possible explanation for the negative relationship between poverty and the probability of visiting a modern health care provider when ill is that there are both direct and indirect costs associated with consulting modern health care providers [20]. These costs could be too substantial for individuals from poor households to bear. The result that poverty negatively affects the demand for modern health care is supported by findings from the literature in three main ways. First, the literature shows that compared to the non–poor, the accessibility of health care services by the poor is low [22,50,51]. Second, according to the literature, the poor tend to use non–modern health care providers such as traditional healers [52] or not seek health care at all [53,54]. Our results actually support this finding from the literature. Third, it is also shown in the literature that the amount of money spent by households on health care services is positively related to household income [55]. There are, however, other studies in the literature that show that income is generally an unimportant determinant of health care provider choice [56].

The lower likelihood of individuals aged between 6 and 14 years, and adults in rural areas compared to those in urban areas visiting a modern health care provider when sick or injured could be due to ease of accessing modern health care facilities in urban areas compared to rural areas [57,58]. This finding is consistent with findings from the literature where, for example, it is reported that in the case of treatment of childhood malaria, caretakers of the children in the rural areas are more likely to resort to self–treatment while their urban counterparts are more likely to take the children for treatment in private or government health care facilities [58]. It is also reported in the literature that in the case of treatment of acute illnesses, more rural residents are more likely to visit faith healers than urban residents [59].

The result that male adults are less likely to seek health care from modern health care providers is, however, not supported by some studies in the literature which show that women are less likely to seek care compared to men [60] and that women and men are equally likely to use medical services [57].

The positive effect of distance to nearest health facility on the demand for modern health care is contrary to expectation. This is because, distance in our health care provider choice models is a proxy for price of modern health care. It actually proxies the indirect cost of modern health care. The positive effect on the probability of visiting a modern health care provider, however, provides evidence of bypassing of nearer facilities to seek health care from farther away facilities [57]. Patients bypass nearer facilities due to mainly price and quality concerns [61,62].

## Conclusion

The main conclusion from this study is that poverty has a negative effect on the demand for modern health care services, other factors held constant. In other words, poor individuals have a less likelihood of consulting modern health care providers when ill compared to their non–poor counterparts, holding other determinants of health care provider choice constant.

Since a major policy objective in most countries regards improvement in peoples’ health through, for example, enabling them to utilize modern health care services when in ill–health, the findings of the study imply that one way of doing this is through the pursuit of strategies that lift people out of poverty. There is massive literature on poverty reduction strategies that countries could pursue (see, for example, [63]). Based on the results from this study, poverty could be reduced by focusing on lowering the average household size (through, for example, increased sensitization on family planning) and lowering distances to modern health care facilities.

## Appendix

In this section we present the detailed results for the health care provider choice models. The results are presented in four tables: Tables 10, 11, 12, and 13. Table 10 shows the results for infants, Table 11 the results for children aged 1 to 5 years, Table 12 the results for children aged 6 to 14 years, and Table 13 the results for adults. In each table we show the results for each provider type for the respective age group. Under each provider type, the results are presented in three columns labelled (1), (2), and (3). The results in column (1) for each provider type are the baseline results. Those in column (2) control for potential endogeneity of poverty status, while those in column (3) control for the potential unobserved heterogeneity of the poverty status.

**Table 10:** **Average marginal effects for health care provider choice models for infants, robust** ***Z*** **statistics in parenthesis**

**Dependent variable = health care provider**									
**None**				**Non–modern**			**Modern**		
**Variable**	**(1)**	**(2)**	**(3)**	**(1)**	**(2)**	**(3)**	**(1)**	**(2)**	**(3)**
Poor	0.051	0.375	0.373	0.002	0.074	0.075	-0.053	-0.449	-0.448
(1.67)		(1.93)	(1.91)	(0.16)	(0.70)	(0.71)	(-1.63)	(-2.10)	(-2.10)
Rural	-0.022	-0.027	-0.027	0.029	0.029	0.028	-0.008	-0.002	-0.001
(-0.64)		(-0.79)	(-0.79)	(1.63)	(1.59)	(1.57)	(-0.21)	(-0.05)	(-0.04)
Male	0.009	0.027	-0.027	0.026	0.029	0.030	-0.035	-0.057	-0.056
(0.31)		(0.87)	(0.85)	(1.72)	(1.79)	(1.80)	(-1.10)	(-1.68)	(-1.67)
Infant age	-0.005	-0.007	-0.007	-0.0009	-0.001	-0.001	0.006	0.008	0.008
(-1.02)		(-1.29)	(-1.29)	(-0.37)	(-0.52)	(-0.51)	(1.12)	(1.44)	(1.44)
Household size	0.011	-0.0002	-0.0001	0.000002	-0.002	-0.003	-0.011	0.003	0.003
(1.89)		(-0.03)	(-0.01)	(0.00)	(-0.48)	(-0.61)	(-1.72)	(0.28)	(0.33)
Fever/Malaria	-0.017	-0.003	-0.003	-0.015	-0.013	-0.013	0.032	0.015	0.016
(-0.50)		(-0.08)	(-0.08)	(-0.93)	(-0.71)	(-0.72)	(0.90)	(0.42)	(0.43)
Flu	0.091	0.081	0.082	0.015	0.013	0.013	-0.107	-0.095	-0.095
(2.30)		(2.02)	(2.02)	(0.84)	(0.71)	(0.72)	(-2.51)	(-2.20)	(-2.21)
Headache	0.392	0.403	0.391	-0.569	-0.564	-0.528	0.177	0.161	0.137
(1.47)		(1.47)	(1.43)	(-6.23)	(-6.18)	(-6.13)	(0.61)	(0.54)	(0.46)
Distance to nearest health facility	0.003	-0.0004	-0.0003	-0.001	-0.002	-0.002	-0.001	0.002	0.002
(1.79)		(-0.17)	(-0.13)	(-2.10)	(-1.59)	(-1.52)	(-0.91)	(0.91)	(0.85)
No education—household head	-0.012	-0.048	-0.048	0.004	-0.003	-0.004	0.008	0.051	0.052
(-0.37)		(-1.22)	(-1.22)	(0.29)	(-0.17)	(-0.22)	(0.23)	(1.22)	(1.23)
Married—household head	-0.059	-0.029	-0.029	0.011	0.018	0.017	0.048	0.011	0.012
(-1.38)		(-0.63)	(-0.63)	(0.49)	(0.76)	(0.72)	(1.05)	(0.21)	(0.24)
Poor residuals		-0.203	-0.207		-0.045	-0.078		0.248	0.285
(-1.67)			(-1.53)	(-0.70)		(-1.25)		(1.87)	(1.97)
Poor interacted with residuals			0.011			0.060			-0.071
			(0.10)			(1.15)			(-0.57)
Number of observations	729	729	729	729	729	729	729	729	729

**Table 11:** **Average marginal effects for health care provider choice models for children aged 1 to 5 years, robust** ***Z*** **statistics in parenthesis**

**Dependent variable = health care provider**									
	**None**			**Non–modern**			**Modern**		
**Variable**	**(1)**	**(2)**	**(3)**	**(1)**	**(2)**	**(3)**	**(1)**	**(2)**	**(3)**
Poor	0.051	0.401	0.409	0.032	0.184	0.181	-0.083	-0.585	-0.590
	(3.21)	(2.99)	(3.03)	(3.93)	(3.05)	(3.00)	(-5.04)	(-4.21)	(-4.21)
Rural	0.0008	-0.008	-0.008	0.044	0.040	0.040	-0.044	-0.032	-0.032
	(0.04)	(-0.43)	(-0.45)	(4.33)	(3.94)	(3.94)	(-2.41)	(-1.73)	(-1.72)
Male	0.008	0.013	0.013	0.007	0.009	0.009	-0.015	-0.023	-0.023
	(0.53)	(0.86)	(0.87)	(0.89)	(1.27)	(1.25)	(-0.93)	(-1.42)	(-1.43)
Household size	0.007	-0.009	-0.009	-0.0008	-0.008	-0.008	-0.006	0.017	0.017
	(2.37)	(-1.30)	(-1.36)	(-0.55)	(-2.55)	(-2.43)	(-1.97)	(2.37)	(2.38)
Fever/Malaria	-0.027	-0.019	-0.018	-0.005	-0.0009	-0.001	0.032	0.020	0.020
	(-1.53)	(-1.04)	(-1.00)	(-0.51)	(-0.09)	(-0.14)	(1.72)	(1.05)	(1.04)
Flu	0.107	0.112	0.113	0.033	0.036	0.035	-0.141	-0.148	-0.148
	(5.20)	(5.45)	(5.46)	(3.36)	(3.55)	(3.52)	(-6.55)	(-6.89)	(-6.89)
Headache	0.018	-0.021	-0.022	0.047	0.030	0.031	-0.065	-0.009	-0.008
	(0.26)	(-0.30)	(-0.32)	(1.75)	(1.08)	(1.11)	(-0.93)	(-0.13)	(-0.12)
Distance to nearest health facility	0.003	0.0005	0.0004	-0.002	-0.003	-0.002	-0.001	0.002	0.002
	(3.78)	(0.39)	(0.30)	(-3.47)	(-4.21)	(-4.01)	(-1.65)	(1.68)	(1.68)
No education—household head	0.040	-0.004	-0.005	0.009	-0.011	-0.010	-0.049	0.015	0.016
	(2.32)	(-0.18)	(-0.22)	(1.05)	(-0.97)	(-0.93)	(-2.71)	(0.61)	(0.62)
Married—household head	-0.011	-0.004	-0.004	-0.013	-0.009	-0.009	0.024	0.013	0.013
	(-0.51)	(-0.20)	(-0.18)	(-1.33)	(-0.90)	(-0.92)	(1.09)	(0.60)	(0.59)
Age	0.017	0.019	0.019	0.005	0.006	0.006	-0.021	-0.025	-0.025
	(3.12)	(3.52)	(3.52)	(1.75)	(2.16)	(2.17)	(-3.82)	(-4.39)	(-4.40)
Poor residuals		-0.217	-0.206		-0.094	-0.110		0.312	0.316
		(-2.64)	(-2.43)		(-2.52)	(-2.82)		(3.64)	(3.59)
Poor interacted with residuals			-0.033			0.035			-0.002
			(-0.56)			(1.15)			(-0.03)
Number of observations	3,306	3,306	3,306	3,306	3,306	3,306	3,306	3,306	3,306

**Table 12:** **Average marginal effects for health care provider choice models for children aged 6 to 14 years, robust** ***Z*** **statistics in parenthesis**

**Dependent variable = health care provider**									
	**None**			**Non–modern**			**Modern**		
**Variable**	**(1)**	**(2)**	**(3)**	**(1)**	**(2)**	**(3)**	**(1)**	**(2)**	**(3)**
Poor	0.083	0.224	0.207	0.033	0.222	0.219	-0.117	-0.447	-0.426
	(5.14)	(2.10)	(1.92)	(3.37)	(3.69)	(3.65)	(-6.99)	(-4.06)	(-3.84)
Rural	0.012	0.010	0.010	0.073	0.070	0.070	-0.085	-0.080	-0.080
	(0.64)	(0.53)	(0.53)	(5.58)	(5.38)	(5.38)	(-4.43)	(-4.19)	(-4.18)
Male	0.004	-0.001	-0.0007	0.008	0.0008	0.001	-0.012	0.0006	-0.0007
	(0.25)	(-0.09)	(-0.05)	(0.85)	(0.08)	(0.15)	(-0.73)	(0.04)	(-0.04)
Household size	0.007	0.002	0.003	-0.002	-0.011	-0.010	-0.005	0.009	0.008
	(2.38)	(0.33)	(0.50)	(-1.34)	(-3.30)	(-3.14)	(-1.46)	(1.63)	(1.36)
Fever/Malaria	-0.001	0.006	0.005	0.014	0.024	0.023	-0.013	-0.030	-0.028
	(-0.07)	(0.32)	(0.27)	(1.24)	(2.02)	(1.97)	(-0.69)	(-1.54)	(-1.46)
Flu	0.156	0.160	0.159	0.056	0.061	0.061	-0.212	-0.221	-0.220
	(6.85)	(6.97)	(6.95)	(4.04)	(4.37)	(4.36)	(-8.88)	(-9.21)	(-9.18)
Headache	0.106	0.097	0.098	0.078	0.065	0.066	-0.184	-0.163	-0.164
	(3.36)	(3.02)	(3.05)	(4.62)	(3.82)	(3.88)	(-5.57)	(-4.84)	(-4.89)
Distance to nearest health facility	0.001	-0.0003	0.00004	-0.003	-0.005	-0.004	0.002	0.005	0.004
	(1.23)	(-0.23)	(0.03)	(-4.37)	(-5.17)	(-4.87)	(2.06)	(3.62)	(3.13)
No education—household head	0.027	0.008	0.010	0.007	-0.018	-0.017	-0.034	0.010	0.007
	(1.55)	(0.37)	(0.46)	(0.67)	(-1.43)	(-1.35)	(-1.86)	(0.42)	(0.29)
Married—household head	-0.015	-0.005	-0.006	-0.020	-0.005	-0.005	0.035	0.010	0.011
	(-0.78)	(-0.24)	(-0.30)	(-1.81)	(-0.38)	(-0.39)	(1.78)	(0.45)	(0.51)
Age	0.059	0.062	0.061	-0.0008	0.003	0.003	-0.058	-0.065	-0.065
	(2.20)	(2.30)	(2.29)	(-0.05)	(0.21)	(0.19)	(-2.09)	(-2.34)	(-2.32)
Age squared	-0.003	-0.003	-0.003	0.0002	-0.0001	-0.00008	0.003	0.003	0.003
	(-2.18)	(-2.31)	(-2.29)	(0.20)	(-0.12)	(-0.10)	(1.98)	(2.30)	(2.27)
Catholic	-0.018	-0.013	-0.014	-0.006	-0.0001	-0.001	0.025	0.013	0.015
	(-0.61)	(-0.41)	(-0.45)	(-0.36)	(-0.01)	(-0.05)	(0.80)	(0.40)	(0.47)
Protestant	-0.018	-0.012	-0.014	-0.0009	0.006	0.006	0.019	0.006	0.008
	(-0.69)	(-0.46)	(-0.53)	(-0.06)	(0.41)	(0.35)	(0.70)	(0.21)	(0.30)
Muslim	-0.048	-0.053	-0.053	-0.045	-0.055	-0.055	0.093	0.108	0.108
	(-1.16)	(-1.27)	(-1.27)	(-1.44)	(-1.80)	(-1.82)	(2.12)	(2.47)	(2.48)
Poor residuals		-0.088	-0.105		-0.118	-0.143		0.206	0.248
		(-1.33)	(-1.53)		(-3.13)	(-3.61)		(3.02)	(3.51)
Poor interacted with residuals			0.056			0.054			-0.110
			(0.98)			(1.56)			(-1.85)
Number of observations	3,523	3,523	3,523	3,523	3,523	3,523	3,523	3,523	3,523

**Table 13:** **Average marginal effects for health care provider choice models for adults (Age ≥ 15 years), robust** ***Z*** **statistics in parenthesis**

**Dependent variable = health care provider**									
	**None**			**Non–modern**			**Modern**		
**Variable**	**(1)**	**(2)**	**(3)**	**(1)**	**(2)**	**(3)**	**(1)**	**(2)**	**(3)**
Poor	0.085	0.271	0.275	0.024	0.156	0.156	-0.109	-0.427	-0.431
	(8.71)	(3.33)	(3.37)	(4.04)	(3.31)	(3.33)	(-10.67)	(-5.02)	(-5.06)
Rural	0.005	0.001	0.002	0.049	0.047	0.046	-0.054	-0.048	-0.048
	(0.49)	(0.13)	(0.16)	(7.02)	(6.61)	(6.56)	(-4.81)	(-4.22)	(-4.21)
Male	0.013	0.016	0.015	0.008	0.010	0.010	-0.021	-0.026	-0.026
	(1.41)	(1.68)	(1.63)	(1.41)	(1.73)	(1.82)	(-2.15)	(-2.59)	(-2.60)
Household size	-0.001	-0.009	-0.009	-0.002	-0.008	-0.008	0.003	0.017	0.017
	(-0.74)	(-2.34)	(-2.37)	(-1.66)	(-3.37)	(-3.41)	(1.65)	(4.10)	(4.16)
Fever/Malaria	-0.00004	0.004	0.005	0.020	0.024	0.024	-0.020	-0.028	-0.028
	(-0.00)	(0.42)	(0.43)	(3.16)	(3.60)	(3.59)	(-1.90)	(-2.56)	(-2.57)
Flu	0.186	0.192	0.192	0.080	0.085	0.085	-0.266	-0.277	-0.277
	(12.14)	(12.37)	(12.36)	(9.29)	(9.60)	(9.65)	(-16.38)	(-16.84)	(-16.86)
Headache	0.082	0.071	0.071	0.076	0.068	0.068	-0.158	-0.139	-0.139
	(4.61)	(3.87)	(3.86)	(7.89)	(6.78)	(6.77)	(-8.43)	(-7.18)	(-7.16)
Distance to nearest health facility	0.003	0.001	0.001	-0.002	-0.003	-0.003	-0.001	0.002	0.002
	(5.77)	(1.44)	(1.26)	(-5.51)	(-5.71)	(-5.45)	(-2.01)	(1.87)	(1.90)
Age	0.001	0.0007	0.0007	-0.0002	-0.0005	-0.0005	-0.0009	-0.0003	-0.0003
	(4.13)	(2.28)	(2.30)	(-1.10)	(-2.47)	(-2.54)	(-3.30)	(-0.79)	(-0.77)
Catholic	-0.107	-0.104	-0.104	0.014	0.017	0.017	0.093	0.087	0.087
	(-7.69)	(-7.43)	(-7.46)	(1.68)	(1.93)	(1.98)	(6.27)	(5.88)	(5.87)
Protestant	-0.089	-0.081	-0.081	0.008	0.014	0.014	0.081	0.067	0.067
	(-6.99)	(-6.15)	(-6.16)	(1.03)	(1.66)	(1.72)	(5.96)	(4.79)	(4.77)
Muslim	-0.065	-0.077	-0.078	-0.017	-0.026	-0.025	0.083	0.103	0.103
	(-3.42)	(-3.90)	(-3.94)	(-1.35)	(-1.92)	(-1.87)	(4.06)	(4.89)	(4.88)
No education	0.014	-0.009	-0.009	0.0005	-0.016	-0.017	-0.015	0.025	0.026
	(1.41)	(-0.63)	(-0.61)	(0.08)	(-1.89)	(-1.96)	(-1.39)	(1.67)	(1.70)
Married	0.001	0.007	0.007	-0.005	-0.001	-0.0007	0.004	-0.006	-0.006
	(0.14)	(0.73)	(0.72)	(-0.95)	(-0.17)	(-0.13)	(0.41)	(-0.60)	(-0.62)
Poor residuals		-0.114	-0.095		-0.081	-0.103		0.196	0.197
		(-2.30)	(-1.82)		(-2.81)	(-3.39)		(3.76)	(3.65)
Poor interacted with residuals			-0.041			0.039			0.002
			(-1.30)			(2.11)			(0.05)
Number of observations	9,694	9,694	9,694	9,694	9,694	9,694	9,694	9,694	9,694

## Additional file

Additional file 1
**This is the questionnaire that was used by the Kenya National Bureau of Statistics to collect the data used in the study.**

## References

[1] World Health Organization (WHO): *World Health Statistics 2013*, Geneva: World Health Organization (WHO); 2013.

[2] Becker GS (2007). **Health as human capital: synthesis and extensions**. Oxford Econ Papers.

[3] Schultz TP (2010). **Health human capital and economic development**. J Afr Economies.

[4] Grossman M (1972). **On the concept of health capital and the demand for health**. J Pol Econ.

[5] Rosenzweig M, Schultz T (1983). **Estimating a household production function: heterogeneity, the demand for health inputs, and their effects on birth weight**. J Pol Econ.

[6] Fuchs V (2004). **Reflections on the socio–economic correlates of health**. J Health Econ.

[7] Mwabu G: **Health economics for low–income countries**. In *Handbook of Development Economics Volume 4*. Edited by Schultz TP, Strauss J. Elsevier/North–Holland: Amsterdam; 2008:3305–3374.

[8] Humphreys B, Mcleod L, Ruseski J (2014). **Physical activity and health outcomes: evidence from Canada**. Health Econ.

[9] Ruebush TK, Kern MK, Campbell CC, Oloo AJ (1995). **Self–treatment of malaria in a rural area of western Kenya**. Bull World Health Org.

[10] Shaikh BT, Hatcher J (2005). **Health seeking behaviour and health service utilization in Pakistan: challenging the policy makers**. J Public Health.

[11] Mbagaya GM, Odhiambo MO, Oniang’o RK (2005). **Mother’s health seeking behaviour during child illness in a rural western Kenyan community**. Afr Health Sci.

[12] Anyanwu JC (2007). **Demand for health care institutions’ services: evidence from malaria fever treatment in Nigeria**. Afr Dev Rev.

[13] Muriithi M, Mwabu G: **Demand for health care in Kenya: the effects of information about quality**. In *Econometric Methods for Analyzing Economic Development*. Edited by Schaeffer P, Kouassi E. Hershey PA: IGI Global; 2013:102–110.

[14] Abubakar A, Van Baar A, Fischer R, Bomu G, Gona J, Newton C (2013). **Socio–cultural determinants of health–seeking behaviour on the Kenyan coast: a qualitative study**. PloS ONE.

[15] Bello R (2005). **Determinants of demand for traditional method of health care services in Osun state: Nigeria**. Ind J Soc Dev.

[16] Andersen R, Newman JF (1973). **Societal and individual determinants of medical care utilization in the United States**. Milbank Memorial Fund Q: Health Soc.

[17] Mwabu G. M (1989). **Nonmonetary factors in the household choice of medical facilities**. Econ Dev Cultural Change.

[18] Ensor T, Cooper S (2004). **Overcoming barriers to health service access: influencing the demand side**. Health Policy Plann.

[19] Victoor A, Delnoij D, Friele R, Rademakers J (2012). **Determinants of patient choice of healthcare providers: a scoping review**. BMC Health Serv Res.

[20] Mwabu G: **The demand for health care**. In *International Encyclopedia of Public Health Volume 2*. Edited by Heggenhougen K, Quah S. San Diego: Academic Press; 2008:84–89.

[21] Farag M, NandaKumar AK, Wallack S, Hodgkin D, Gaumer G, Erbil C (2012). **The income elasticity of health care spending in developing and developed countries**. Int Jo Health Care Finance Econ.

[22] Peters DH, Garg A, Bloom G, Walker DG, Brieger WR, Rahman MH (2008). **Poverty and access to health care in developing countries**. Ann N Y Acad Sci.

[23] Xu K, Evans DB, Kawabata K, Zeramdini R, Klavus J, Murray C (2003). **Household catastrophic health expenditure: a multicountry analysis**. Lancet.

[24] Wagstaff A, Van Doorslaer E (2003). **Catastrophe and impoverishment in paying for health care: with applications to Vietnam 1993–1998**. Health Econom.

[25] Chuma J, Maina T (2012). **Catastrophic health care spending and impoverishment in Kenya**. BMC Health Serv Res.

[26] Vella F (1998). **Estimating models with sample selection bias: a survey**. J Hum Resour.

[27] Stock JH, Watson MW: *Introduction to Econometrics*, Boston: Addison-Wesley; 2011.

[28] Cameron AC, Trivedi PK: *Microeconometrics Using Stata. College Station*, Texas: Stata Press; 2010.

[29] Terza JV, Basu A, Rathouz PJ (2008). **Two-stage residual inclusion estimation: addressing endogeneity in health econometric modelling**. J Health Econ.

[30] Gourieroux C, Monfort A, Renault E, Trognon A (1987). **Generalized residuals**. J Econometrics.

[31] Bollen KA, Guilkey DK, Mroz TA (1995). **Binary outcomes and endogenous explanatory variables: tests and solutions with an application to the demand for contraceptive use in Tunisia**. Demography.

[32] Zohoori N, Savitz DA (1997). **Econometric approaches to epidemiologic data: relating endogeneity and unobserved heterogeneity to confounding**. Ann Epidemiol.

[33] Florens JP, Heckman JJ, Meghir C, Vytlacil E (2008). **Identification of treatment effects using control functions in models with continuous, endogenous treatment and heterogeneous effects**. Econometrica.

[34] Brookhart MA, Rassen JA, Schneeweiss S (2010). **Instrumental variable methods in comparative safety and effectiveness research**. Pharmacoepidemiology Drug Safety.

[35] Petrou S, Kupek E (2010). **Poverty and childhood undernutrition in developing countries: a multi-national cohort study**. Soc Sci Med.

[36] Long JS: *Regression Models for Categorical and Limited Dependent Variables*, Thousand Oaks: Sage Publications; 1997.

[37] Amemiya T (1981). **Qualitative response models: a survey**. J Econ Lit.

[38] Wooldridge J: *Econometric Analysis of Cross Section and Panel Data*. 2nd edn, Cambrdige, Massachussetts: MIT Press; 2010.

[39] Kenya National Bureau of Statistics (KNBS): *Kenya Integrated Household Budget Survey (KIHBS) 2005/06: Basic Report*, Nairobi: Kenya National Bureau of Statistics (KNBS); 2007.

[40] Kenya National Bureau of Statistics (KNBS): *Basic Report on Well-being in Kenya: Based on Kenya Integrated Household Budget Survey 2005/06*, Nairobi: Kenya National Bureau of Statistics (KNBS); 2007.

[41] Geda A, de Jong N, Kimenyi M, Mwabu G: **Determinants of poverty in Kenya: a household level analysis**. *Economics Working Papers*2005:2005–44. [http://digitalcommons.uconn.edu/econ_wpapers/200544]

[42] Anyanwu JC (2011). **Towards reducing poverty in Nigeria: the case of Igboland**. J Econ Int Finance.

[43] Anyanwu JC (2014). **Marital status, household size and poverty in Nigeria: evidence from the 2009/2010 survey data**. Afr Dev Rev.

[44] Anyanwu JC (2013). **The correlates of poverty in Nigeria and policy implications**. Afr J Econ Sustainable Dev.

[45] Paudel R, Upadhyaya T, Pahari D (2012). **People’s perspective on access to health care services in a rural district of Nepal**. J Nepal Med Assoc.

[46] Khan MM, Hotchkiss D, Berruti A, Hutchinson P (2006). **Geographic aspects of poverty and health in Tanzania: does living in a poor area matter?**. Health Policy Plan.

[47] Okwi PO, Ndeng’e G, Kristjanson P, Arunga M, Notenbaert A, Omolo A, Henninger N, Benson T, Kariuki P, Owuor J: **Spatial determinants of poverty in rural Kenya**. In *Proceedings of the National Academy of Sciences of the United States*. Edited by Dasgupta PS. Washington, DC: PNAS; 2007:16769–16774. 10.1073/pnas.0611107104 PMC2040447 17942704

[48] McLaren Z, Ardington C, Leibbrandt M: **Distance as a barrier to health care access in South Africa**. *Southern Africa Labour and Development Research Unit Working Paper 97.*2013. [www.opensaldru.uct.ac.za/handle/11090/613]

[49] Kimenyi M, Mwabu G, Manda D (2006). **Human capital externalities and private returns to education in Kenya**. Eastern Econ J.

[50] Borghi J, Ensor T, Somanathan A, Lissner C, Mills A (2006). **Mobilising financial resources for maternal health**. Lancet.

[51] Mebratie A, Van de Poel E, Yilma Z, Abebaw D, Alemu G, Bedi A (2014). **Healthcare–seeking behaviour in rural Ethiopia: evidence from clinical vignettes**. BMJ Open.

[52] Onwujekwe O, Uzochukwu B, Obikeze E, Okoronkwo I, Ochonma O, Onoka C, Madubuko G, Okoli C (2010). **Investigating determinants of out–of–pocket spending and strategies for coping with payments for healthcare in southeast Nigeria**. BMC Health Serv Res.

[53] Amaghionyeodiwe L (2008). **Determinants of the choice of health care provider in Nigeria**. Health Care Manag Sci.

[54] Habtom G, Ruys P (2007). **The choice of a health care provider in Eritrea**. Health Policy.

[55] Ogundari K, Abdulai A (2014). **Determinants of household’s education and healthcare spending in Nigeria: evidence from survey data**. Afr Dev Rev.

[56] Lindelow M (2005). **The utilisation of curative healthcare in Mozambique: does income matter?**. J Afr Econ.

[57] Mwabu G, Wang’ombe J, Nganda B (2003). **The demand for medical care in Kenya**. Afri Dev Rev.

[58] Okeke T, Okeibunor J (2010). **Rural–urban differences in health–seeking for the treatment of childhood malaria in south–east Nigeria**. Health Policy.

[59] Shah T, Patel M, Shah V (2013). **Health care seeking behaviour of urban and rural community in Ahmedabad district**. Int Jo Med Sci Publ Health.

[60] Ahmed S, Adams A, Chowdhury M, Bhuiya A (2000). **Gender, socioeconomic development and health–seeking behaviour in Bangladesh**. Soc Sci Med.

[61] Varkevisser M, Van der Geest S (2007). **Why do patients bypass the nearest hospital? an empirical analysis for orthopaedic care and neurosurgery in the Netherlands**. Eur J Health Econ.

[62] Gauthier B, Wane W (2011). **Bypassing health providers: the quest for better price and quality of health care in Chad**. Soc Sci Med.

[63] *World Development Report 2000/2001: Attacking Poverty*, New York: World Bank; 2001.

