# Supplementary material for: Poverty and health care demand in Kenya
Source: BMC Health Serv Res. 2014 Nov 22;14:560. doi: 10.1186/s12913-014-0560-y (PMC4243287; doi:10.1186/s12913-014-0560-y)
Supplement: Additional file 1 — This is the questionnaire that was used by the Kenya National Bureau of Statistics to collect the data used in the study. [file 12913_2014_560_MOESM1_ESM.pdf]

STRICTLY CONFIDENTIAL

QUESTIONNAIRE SERIAL NO.

CLUSTER					
HOUSEHOLD NUMBER					
HOUSEHOLD SERIAL NO.					

GPS CO-ORDINATES

	Degrees	*Min*	*Sec*
LATITUDE			
LONGITUDE			
ALTITUDE M.			

CENTRAL BUREAU OF STATISTICS

Ministry of Planning and National Development
P. O. Box 30266-00100 Nairobi. KENYA
Tel. No. 317612/22/23: Email: herufi@cbs.go.ke

KENYA INTEGRATED HOUSEHOLD BUDGET SURVEY (KIHBS), 2004/05
HOUSEHOLD QUESTIONNAIRE

THIS SURVEY IS BEING CONDUCTED BY THE CENTRAL BUREAU OF STATISTICS AS MANDATED BY THE STATISTICS ACT (CAP 112 OF THE LAWS OF KENYA).
THIS INFORMATION IS STRICTLY CONFIDENTIAL AND IS TO BE USED FOR STATISTICAL PURPOSES ONLY.

SECTION A-1: HOUSEHOLD IDENTIFICATION

WRITE CODES and NAMES for Province; District; Division; Location; Sub-location; Cluster No.; Dwelling Structure No.;Household No. and Household Head.

		CODE	NAME
A01.	PROVINCE		
A02.	DISTRICT		
A03.	DIVISION		
A04.	LOCATION		
A05.	SUB-LOCATION		
A06.	CLUSTER		
A07.	HOUSEHOLD NUMBER		
A08.	DWELLING STRUCTURE NO.:		
A09.	LANGUAGE OF INTERVIEW		

01	EMBU	06	LUHYA	11	SOMALI
02	KALENJIN	07	LUO	12	SWAHILI
03	KAMBA	08	MAASAI	13	ENGLISH
04	KIKUYU	09	MERU		
05	KISII	10	MIJIKENDA		

IF YOU USE MORE THAN ONE QUESTIONNAIRE TO COLLECT INFORMATION FROM THIS HOUSEHOLD, INDICATE BELOW e.g. 1 of 2.

[ ] of [ ]

A10. NAME OF HOUSEHOLD HEAD: ____________________

A11. DOES THIS HOUSEHOLD REPLACE ANOTHER SAMPLE HOUSEHOLD CHOSEN FOR THE SURVEY? YES..1; NO..2 **(»A14)**

A12. WHICH HOUSEHOLD IN THIS CLUSTER DOES IT REPLACE? HOUSEHOLD NUMBER OF ORIGINALLY SELECTED HOUSEHOLD

A13. WHY WAS ORIGINALLY SELECTED HOUSEHOLD REPLACED?

1 - DWELLING FOUND, BUT NO HH MEMBER COULD BE FOUND.
2 - DWELLING FOUND, BUT RESPONDENT REFUSED.
3 - DWELLING FOUND, BUT UNOCCUPIED.
4 - DWELLING FOUND, BUT NOT A RESIDENTIAL BUILDING.
5 - DWELLING DESTROYED.
6 - DWELLING NOT FOUND.

SECTION A-2: SURVEY STAFF DETAILS

A14. NAME OF INTERVIEWER: ______________________

A15. INTERVIEWER CODE: | | | |

RESULT CODES
1 COMPLETED.
2 NO ONE HOME
3 PARTIAL, INCOMPLETE.

A16. DATE OF INTERVIEW:

	DD / MM / YY	SECTIONS	RESULT
First visit	/ /		
Second visit	/ /		
Third visit	/ /		
Forth visit	/ /		
Fifth visit	/ /		
Sixth visit	/ /		
Seventh visit	/ /		
Eighth visit	/ /		
Ninth visit	/ /		
Tenth visit	/ /		

A28. DATE OF DATA ENTRY:

	DD / MM / YY	SECTIONS	RESULT
First Entry	/ /		
Second Entry	/ /		
Third Entry	/ /		
Forth Entry	/ /		
Fifth Entry	/ /		
Sixth Entry	/ /		
Seventh Entry	/ /		
Eighth Entry	/ /		
Ninth Entry	/ /		
Tenth Entry	/ /		

A17. NAME OF TEAM LEADER: ______________________

A18. TEAM LEADER CODE:

A19. DATE OF QUESTIONNAIRE INSPECTION:

A20. NAME OF ZONE COORDINATOR

A21. ZONE COORDINATOR CODE

A22. DATE OF INSPECTION

A23. NAME OF DATA ENTRY OPERATOR IN FIELD:

A24. DATA ENTRY OPERATOR CODE:

A25. NAME OF DATA ENTRY OPERATOR AT HQ:

A26. DATA ENTRY OPERATOR CODE:

A27. DATE OF DATA VALIDATION:

SPECIAL REMARKS/COMMENTS

INTRODUCTION TO THE HOUSEHOLD TO BE INTERVIEWED

CONVEY THE FOLLOWING INFORMATION TO THE RESPONDENT

The Central Bureau of Statistics occasionally selects at random several hundred households in the country to ask them questions about how they live. The responses provided by the households help the Government of Kenya and other key stakeholders to plan for the welfare of all Kenyans.

Your household was randomly selected as one of those to which the KIHBS questions will be asked from a list of all of the households in this area.

I therefore, would like to ask you some questions as a responsible member of this household. I will also need your assistance in asking some questions to other members of your household; weighing and measuring the height of any children under age 5 years who live in your household;and obtaining measurement of your rooms. These questions will take some time to complete and therefore I would appreciate your patience. I want to assure you that under no circumstance will the information be used for any purpose other than meeting the objectives of the survey. I shall also be visting your house frequently for the next 20 days
Before I start, do you have any questions or is there anything which I have said on which you would like any further clarification? Otherwise, may I proceed with interviewing you and members of your household?

Tick appropriately ☐ ACCEPT ☐ REJECT

TABLE OF CONTENTS

Page		Page	
1	SECTION A-1: HOUSEHOLD IDENTIFICATION	37	SECTION K: NON FOOD ITEMS EXPENDITURES - PAST ONE MONTH
2	SECTION A-2: SURVEY STAFF DETAILS	39	SECTION L: NON-FOOD EXPENDITURES – PAST ONE MONTH
4	SECTION B: HOUSEHOLD MEMBER ROSTER	41	SECTION M: EXPENDITURES ON DURABLES
6	SECTION C: EDUCATION	44	SECTION N: AGRICULTURE HOLDING
9	SECTION D: HEALTH, FERTILITY AND HOUSEHOLD DEATHS	47	SECTION O: AGRICULTURE OUTPUT
14	SECTION E: LABOUR	49	SECTION P: LIVESTOCK
18	SECTION F: CHILD HEALTH AND ANTHROPOMETRY	53	SECTION Q: HOUSEHOLD ENTERPRISES
23	SECTION G: HOUSING	55	SECTION R: TRANSFERS
24	SECTION H: WATER, SANITATION AND ENERGY USE	56	SECTION S: OTHER INCOME
26	SECTION H: WATER, SANITATION AND ENERGY USE	57	SECTION T: RECENT SHOCKS TO HOUSEHOLD WELFARE
27	SECTION I: CONSUMPTION OF FOOD OVER PAST ONE WEEK	58	SECTION U: CREDIT
35	SECTION J: REGULAR NON FOOD ITEMS EXPENDITURES - PAST ONE MONTH		

IN ORDER TO MAKE A COMPREHENSIVE LIST OF INDIVIDUALS CONNECTED TO THE HOUSEHOLD, USE THE FOLLOWING PROBE QUESTIONS:

First, give me the names of all the members of your immediate (nuclear) family who normally live and eat their meals together here.

WRITE DOWN NAMES, SEX, AND RELATIONSHIP TO HOUSEHOLD HEAD (B02 to B04). LIST HOUSEHOLD HEAD ON LINE 1.

Then, give me the names of any other persons related to you or other household members who normally live and eat their meals together here.

FILL IN B02 to B04.

Are there any other persons not here now who normally live and eat their meals here? For example, household members studying elsewhere or traveling.

FILL IN B02 to B04.

Then, give me the names of any other persons not related to you or other household members, but who normally live and eat their meals together here, such as live-in servants.

FILL IN B02 to B04.

Finally, are there any other persons who slept here last night, but who do not normally live here such as visitors, persons on transit?

FILL IN B02 to B04.

IF MORE THAN 12 INDIVIDUALS, USE SECOND QUESTIONNAIRE. MAKE SURE TO MARK BOX ON FIRST PAGE OF BOTH QUESTIONNAIRES.

SECTION B: HOUSEHOLD MEMBER ROSTER

[ASK DIRECTLY OF ALL PERSONS IN THE HOUSEHOLD. MOTHERS OR GUARDIANS TO ANSWER FOR CHILDREN UNDER 10 YEARS OF AGE.]

B01<br>ID CODE	B02<br>NAME<br><br>MAKE A COMPLETE LIST OF ALL INDIVIDUALS WHO NORMALLY LIVE AND EAT THEIR MEALS TOGETHER IN THIS HOUSEHOLD, STARTING WITH THE HEAD OF HOUSEHOLD.<br><br>(CONFIRM THAT HOUSEHOLD HEAD HERE IS SAME AS HOUSEHOLD HEAD LISTED ON COVER.)	B03<br>What is [NAME]'s relationship to the head of household?<br><br>HEAD 1<br>SPOUSE 2<br>SON 3<br>DAUGHTER 4<br>FATHER/MOTHER 5<br>SISTER/BROTHER 6<br>GRANDCHILD 7<br>OTHER RELATIVE (SPECIFY) 8<br>SERVANT (live-in)<br>OTHER NON-RELATIVE 9<br>(SPECIFY) 10		B04<br>SEX<br><br>MALE 1<br>FEMALE 2		B05<br>How old is [NAME]?<br><br>IF 6 YEARS OR OLDER, GIVE YEARS ONLY. IF LESS THAN 6 YEARS IN AGE, GIVE YEARS AND MONTHS.<br><br>97 YEARS AND OVER, CODE 97. AGE IS NOT KNOWN, CODE 98.<br>NOT STATED, CODE 99.<br><br>YEARS	MONTHS
1						___ ___	___ ___
2						___ ___	___ ___
3						___ ___	___ ___
4						___ ___	___ ___
5						___ ___	___ ___
6						___ ___	___ ___
7						___ ___	___ ___
8						___ ___	___ ___
9						___ ___	___ ___
10						___ ___	___ ___
11						___ ___	___ ___
12						___ ___	___ ___

B01	B06			B07		B08	B09	B10				B11	B12
I<br>D<br><br>C<br>O<br>D<br>E	What is NAME's date of birth?<br><br>D K 98<br>NS 99			For how many cumulated months during the last 12 months (since MONTH/YEAR) has [NAME] been away from this household? FOR VISITORS CODE 77 and TERMINATE INTERVIEW		WHICH HOUSEHOLD MEMBER PROVIDED INFORMATIO N OF THE INDIVIDUAL?<br><br>WRITE ID CODE	Where was NAME raised/brought up?<br><br>CURRENT VILLAGE /TOWN/CITY 1 (»B13)<br>OTHER VILLAGE IN THIS DISTRICT 2 (»B13)<br>OTHER TOWN/CITY IN THIS DISTRICT 3 (»B13)<br>VILLAGE IN OTHER DISTRICT 4<br>TOWN/CITY IN OTHER DISTRICT 5<br>OUTSIDE KENYA 6	What was NAME's District/Country of birth?<br><br>WRITE DISTRICT or COUNTRY	CODE BY SUPERVISOR			In which year did NAME move to this district or country? ENTER 4 DIGITS<br><br>DK=9998	Why did NAME move to this district/country?<br><br>PARENTS MOVED . . . . 1<br>TO LIVE WITH RELATIVES 2<br>SCHOOLING . . . . 3<br>MARRIAGE . . . . 4<br>FAMILY QUARREL . . . . 5<br>DIVORCE . . . . 6<br>RETURN FROM WORK ELSEWHERE . . . . 7<br>JOB TRANSFER . . . . 8<br>LOOK FOR WORK . . . . 9<br>START NEW JOB OR BUSINESS . . . . 10<br>LOOKING FOR LAND TO FARM . . . . 11<br>TO RECOVER FROM ILLNESS 12<br>OTHER (*SPECIFY*) 13
	DAY	MONTH	YEAR	CUMULATED MONTHS					Province	District		YEAR	
1													
2													
3													
4													
5													
6													
7													
8													
9													
10													
11													
12													

	For persons aged 30 years and below														
B01	B13	B14	B15	B16	B17	B18	B19	B20	B21				B22	B23	B24
ID CODE	Is NAME's biological father alive?<br><br>YES, LIVING IN THIS HOUSEHOLD, COPY ROSTER ID CODE<br>(»B15)<br>YES, LIVING OUTSIDE THIS HOUSEHOLD . . . . 96<br>(»B15)<br>DECEASED . . . . 97<br>DOESN'T KNOW . . . . . . 98<br>(»B15)	What was NAME's age when father died?<br><br>D K 98<br>NS 99<br><br>AGE IN COMPLETED YEARS	Is NAME'S biological mother alive?<br><br>YES, LIVING IN THIS HOUSEHOLD,<br>COPY ROSTER ID CODE<br>(»B17)<br>YES LIVING OUTSIDE THIS HOUSEHOLD . . . . 96<br>(»B17)<br>DECEASED . . . . 97<br>DOESN'T KNOW . . . . . 98<br>(»B17)	What was NAME's age when mother died?<br><br>D K 98<br>NS 99<br><br>AGE IN COMPLETED YEARS	CHECK: IS THE RESPONDENT 10 YEARS AND OLDER?<br><br>YES . . . . 1<br>NO . . . . . 2<br>»NEXT PERSON	What is NAME's religion?<br><br>CATHOLIC 1<br>PROTESTANT 2<br>OTHER CHRISTIAN 3<br>MUSLIM 4<br>TRADITIONALIST 5<br>NO RELIGION 6<br>OTHER RELIGION 7	What is NAME's marital status?<br><br>MONOGAMOUS MARRIED . . . . . . 1<br>POLYGAMOUS MARRIED . . . . . . 2<br>LIVING TOGETHER . . . . 3<br>SEPARATED . . . . 4<br>DIVORCED . . . . . . 5<br>WIDOW OR WIDOWER . . . . . . 6<br>NEVER MARRIED . . . . . . 7<br>If Code 4,5,6,or 7 »B24	Does spouse/ partner live in this household now?<br><br>YES . . . . 1<br>NO. . . . . 2<br>(»B22)	COPY THE ID CODE OF THE SPOUSE/PARTNER.<br><br>IF MORE THAN ONE SPOUSE, COPY ID CODES OF ALL SPOUSES RESIDENT IN HOUSEHOLD FROM ROSTER<br><br>(THEN, IF FEMALE »B24. IF MALE »B23.)				Does NAME have a spouse living outside of this house-hold?<br><br>YES . . . . 1<br>NO . . . . 2<br>IF NO >>B24	How many spouses does NAME have who are residing outside of this household?<br><br>NUMBER	ASK HH HEAD OR ANY RESPONSIBLE MEMBER OF HH:<br>How many children aged under 15 years who are children of an adult in this household <u>live elsewhere</u> (live outside household)?<br><br>NUMBER
									FIRST	SECOND	THIRD	FOURTH			
1															
2															
3															
4															
5															
6															
7															
8															
9															
10															
11															
12															

SECTION C: EDUCATION

[ASK ALL PERSONS AGED 3 YEARS AND OLDER. MOTHERS OR GUARDIANS TO ANSWER FOR CHILDREN UNDER 10 YEARS OF AGE.]

C01	C02	C03	C04		C05	C06	C07	C08	C09	C10
I<br>D<br><br>C<br>O<br>D<br>E	PUT CODE '1' FOR ALL INDIVIDUALS WHO ARE AGED UNDER 3 YEARS. OTHERWISE CODE 2.<br><br>DO NOT ADMINISTER THIS MODULE TO THESE INDIVIDUALS CODED 1.	Has NAME ever attended school?<br><br>Yes . . . . 1<br>NO . . . . 2<br>(»C11)	What is the highest school grade that NAME has completed and in which year?<br><br>PRE -SCHOOL.. .00 (»C06)<br>**PRIMARY**<br>STD. 1 . . . . .01<br>STD. 2 . . . . .02<br>STD. 3 . . . . .03<br>STD. 4 . . . . .04<br>STD. 5 . . . . .05<br>STD. 6 . . . . .06<br>STD. 7 . . . . .07<br>STD. 8 . . . . .08<br>**SECONDARY**<br>FORM 1 . . . . .09<br>FORM 2 . . . . .10<br>FORM 3 . . . . .11<br>FORM 4 . . . . .12<br>FORM 5 . . . . .13<br>FORM 6 14	**UNIVERSITY**<br>UNIV. 1 . . . . 15<br>UNIV. 2 . . . . 16<br>UNIV. 3 . . . . 17<br>UNIV. 4 . . . . 18<br>UNIV. 5 &<br>ABOVE . . . . 19<br>None 20<br>Other specify 21	What was NAME highest vocational training completed?<br><br>GOVERNMENT<br>COLLEGE . . . . 01<br>COMMERCIAL<br>COLLEGE . . . . 02<br>VOCATIONAL/<br>VILLAGE . . . . 03<br>NONE. 04	What is the highest educational qualification has NAME acquired?<br><br>NONE . . . . 01<br>CPE/KCPE . . . . 02<br>KCE/KCSE . . . . 03<br>KJSE . . . . .04<br>KACE/EAACE . . . . .05<br>CERTIFICATE . . . . 06<br>PUBLIC INSTITUTION<br>DIPLOMA . . . . .07<br>PRIVATE INSTITUTION<br>DIPLOMA,.................. 08<br>DEGREE . . . . 09<br>POST-GRAD.UATE<br>DEGREE /DIPLOMA . . . 10<br>OTHER SPECIFY. 11	How old was NAME when started school?	Did NAME attend school/ academic institution in the last school year?<br><br>YES . . . . 1<br>NO . . . . 2<br>(»C10)	What grade was NAME attending last school year?<br><br>PRE-SCHOOL . . 00<br>**PRIMARY**<br>STD. 1 . . . . 01<br>STD. 2 . . . . 02<br>STD. 3 . . . . 03<br>STD. 4 . . . . 04<br>STD. 5 . . . . 05<br>STD. 6 . . . . 06<br>STD. 7 . . . . 07<br>STD. 8 . . . . 08<br>**SECONDARY**<br>FORM 1 . . . . . . .09<br>FORM 2 . . . . 10<br>FORM 3 . . . . 11<br>FORM 4 . . . . 12<br>FORM 5 . . . . 13<br>FORM 6 . . . . 14<br>**UNIVERSITY**<br>UNIV. 1 . . . . .15<br>UNIV. 2 . . . . .16<br>UNIV. 3 . . . . .17<br>UNIV. 4 . . . . .18<br>UNIV. 5 &<br>ABOVE . . . . . . .19<br>**TRAINING**<br>GOVERNMENT<br>COLLEGE . . . . . 20<br>COMMERCIAL<br>COLLEGE . . . . . 21<br>VOCATIONAL/<br>VILLAGE . . . . . . 22<br>OTHER 23	Is NAME currently attending school?<br><br>IF SCHOOL IS NOT IN SESSION NOW, ASK: Did you attend school in the session just completed and plan to attend next session?<br><br>YES . . . . 1<br>(»C12)<br>NO 2
			GRADE	YEAR			YEARS			
1										
2										
3										
4										
5										
6										
7										
8										
9										
10										
11										
12										

C01	C11		C12	C13	C14	C15	C16	C17	C18
ID CODE	Why did NAME stop or never attend school?<br>STILL TOO YOUNG<br>TO ATTEND SCHOOL . . . . . 00<br>NO MONEY FOR<br>SCHOOL COSTS . . . . . 01<br>POOR QUALITY OF SCHOOLS . . . . . 02<br>OWN ILLNESS/DISABILITY . . . . . 03<br>FAMILY ILLNESS/DISABILITY . . . . . 04<br>NOT INTERESTED, LAZY . . . . . 05<br>PARENTS DID NOT LET ME . . . . . 06<br>HAD TO WORK OR HELP AT HOME . . . . . 07<br>SCHOOL TOO FAR<br>FROM HOME . . . . . 09<br>SCHOOL CONFLICT WITH<br>BELIEFS . . . . . 10<br>OTHER (SPECIFY) . . . . . 11<br>(»C16)		What is the current grade NAME is attending?<br>PRE-SCHOOL . . . . . 00<br>**PRIMARY**<br>STD. 1 . . . . . 01<br>STD. 2 . . . . . 02<br>STD. 3 . . . . . 03<br>STD. 4 . . . . . 04<br>STD. 5 . . . . . 05<br>STD. 6 . . . . . 06<br>STD. 7 . . . . . 07<br>STD. 8 . . . . . 08<br>**SECONDARY**<br>FORM 1 . . . . . 09<br>FORM 2 . . . . . 10<br>FORM 3 . . . . . 11<br>FORM 4 . . . . . 12<br>FORM 5 . . . . . 13<br>FORM 6 . . . . . 14<br>**UNIVERSITY**<br>UNIV. 1 . . . . . 15<br>UNIV. 2 . . . . . 16<br>UNIV. 3 . . . . . 17<br>UNIV. 4 . . . . . 18<br>UNIV. 5 &<br>ABOVE . . . . . 19<br>**TRAINING**<br>GOVERNMENT<br>COLLEGE . . . . . 20<br>COMMERCIAL<br>COLLEGE . . . . . 21<br>VOCATIONAL/<br>VILLAGE . . . . . 22<br>OTHER SPECIFY 23	Who runs/ manages school NAME attending?<br>GOVERNMENT . . . . . 01<br>PRIVATE CHURCH . . . . . 02<br>PRIVATE MUSLIM . . . . . 03<br>PRIVATE OTHER . . . . . 04<br>COMMUNITY . . . . . 05<br>OTHER 09	How many days was NAME's school in session over the <u>past 2 weeks</u>?<br>NUMBER	How many days did NAME attend school in the <u>past 2 weeks</u>?<br>NUMBER	CHECK: IF C08=YES or C10=YES<br>**CODE YES .1**<br>**otherwise code 2**<br>**and » C24**	Did NAME at any time in the past 12 months, <u>temporarily withdraw from school</u>, so that NAME missed more than 2 consecutive weeks of instruction?<br>Yes . . . . . 1<br>NO . . . . . 2<br>(»C19)	What was the **Main Reason** for NAME <u>temporarily withdrawing from school</u>?<br>STILL TOO YOUNG<br>TO ATTEND SCHOOL . . . . . 00<br>NO MONEY FOR<br>SCHOOL COSTS . . . . . 01<br>POOR QUALITY OF SCHOOLS . . . . . 02<br>OWN ILLNESS/DISABILITY . . . . . 03<br>FAMILY ILLNESS/DISABILITY . . . . . 04<br>NOT INTERESTED, LAZY . . . . . 05<br>PARENTS DID NOT LET ME . . . . . 06<br>HAD TO WORK OR HELP AT HOME . . 07<br>SCHOOL TOO FAR<br>FROM HOME . . . . . 09<br>SCHOOL CONFLICT WITH<br>BELIEFS . . . . . 10<br>OTHER (SPECIFY) 11
	1st Reason	2nd Reason							
1									
2									
3									
4									
5									
6									
7									
8									
9									
10									
11									
12									

C01<br><br>I<br>D<br><br>C<br>O<br>D<br>E	C19<br>How much was spent on NAME's education in the last 12 months by members of your household:<br><br>IF NOTHING WAS SPENT, WRITE ZERO.												C20<br>Did any persons outside your household contribute to NAME'S education expenses?	C21<br>What was the value of this assistance received in the past 12 months?<br><br>INCLUDE VALUE OF IN KIND	C22<br>Did NAME receive a scholarship to help pay for your educational expenses in the past 12 months?	C23<br>What was the value of this assistance received in the past 12 months?<br><br>INCLUDE VALUE OF IN KIND	C24<br>Can NAME read in any language?	C25<br>Can NAME write in any language?
	A.<br>Tuition fees<br>KSHS	B.<br>Books & other materials<br>KSHS	C.<br>Uniform including other clothing<br>KSHS	D.<br>Boarding fees<br>KSHS	E<br>Transport costs<br>KSHS	F.<br>Contribution for school building or maintanance<br>KSHS	G.<br>Extra tuition fees<br>KSHS	H.<br>Examination fees<br>KSHS	I.<br>PTA & other related fees<br>KSHS	J.<br>Pocket Money & Shopping<br>KSHS	K.<br>Other Expenses<br>KSHS	L.<br>Total<br>KSHS	YES . . . . . . 1<br>NO . . . . 2<br>(»C22)	KSHS	YES . . . . 1<br>NO . . . . 2<br>(»C24)	KSHS	CANNOT<br>READ AT ALL. . . . 1<br>CAN READ<br>PART OF SENTEN 2<br>CAN READ<br>WHOLE SENTENC 3<br>NO SENTENCE<br>IN REQUIRED LAN 4	YES . . . . . . 1<br>NO 2
1																		
2																		
3																		
4																		
5																		
6																		
7																		
8																		
9																		
10																		
11																		
12																		

SECTION D: HEALTH, FERTILITY AND HOUSEHOLD DEATHS

[ASK OF ALL PERSONS IN THE HOUSEHOLD. MOTHERS OR GUARDIANS TO ANSWER FOR CHILDREN UNDER 10 YEARS OF AGE.]

D01	D02	D03	D04	D05		D06	D07	D08	D09		D10	D11
I D CODE	ID NO. OF PERSON REPORTING THE INFORMATION FOR THE INDIVIDUAL	Was NAME sick or injured in the last 4 weeks?	Was NAME's sickness / injury work related?	What sort of sickness/injury did NAME suffer from?<br>FEVER, MALARIA .... 01<br>DIARRHEA 02<br>STOMACH ACHE 03<br>VOMITING 04<br>UPPER RESPIRATORY(SINUSES) 05<br>LOWER RESPIRATORY (CHEST, LUNGS 06<br>FLU 07<br>ASTHMA 08<br>HEADACHE 09<br>SKIN PROBLEM 10<br>DENTAL PROBLEM 11<br>EYE PROBLEM 12<br>EAR/NOSE/THROAT 13<br>BACKACHE 14<br>HEART PROBLEM 15<br>BLOOD PRESSURE 16<br>PAIN WHEN PASSING URINE 17<br>DIABETES 18<br>MENTAL DISORDER 19<br>TB 20	SEXUALLY TRANSMITTED DISEAS 21<br>BURN 22<br>FRACTURE 23<br>WOUND 24<br>POISONING 25<br>PREGNANCY RELATED 26<br>UNSPECIFIED LONG-TERM ILLNE: 27<br>HIV/AIDS 28<br>TYPHOID 29<br>OTHER (SPECIFY) 30	Who diagnosed the illness?<br>MEDICAL WORKER (DOCTOR, CLINICAL OFFICER, NURSE) AT HOSPITAL 1<br>MEDICAL WORKER AT OTHER HEALTH FACILITY 2<br>TRADITIONAL HEALER 3<br>NON-HH MEMBER (NOT MEDICAL) 4<br>HH MEMBER 5<br>SELF 6<br>HERBALIST 7<br>FAITH HEALER 8<br>OTHERS .(specify) 9	How many days of work/school did NAME miss due to illness/injury in the last 4 weeks? **FOR PERSONS 3YRS AND ABOVE**<br>N/A 99	Did NAME consult a health provider on these sicknesses /injury in the last 4 weeks?<br>YES. . . . . 1<br>NO 2	What kind of health provider did NAME visit? UP TO TWO VISITS BY ORDER OF PROBLEM.<br>REFERAL HOSPITAL 01<br>DISTRICT/PROVINCIAL/HOSPITAL 02<br>PUBLIC DISPENSARY 03<br>PUBLIC HEALTH CENTER 04<br>PRIVATE DISPENSARY/ HOSPITAL 05<br>PRIVATE CLINIC 06<br>TRADITIONAL HEALER 07<br>MISSIONARY HOSP./DISP 08<br>PHARMACY/CHEMIST 09<br>KIOSK 10<br>FAITH HEALER 11<br>HERBALIST 12<br>OTHER (SPECIFY) 13		How many times did NAME use any health service due to sickness/injury in the last 4 weeks?	Did NAME visit a health provider for any other health related reason (not sick) in the last 4 weeks?<br>YES. . . . . . . 1<br>NO 2
	WRITE MEMBER ID CODE	YES. . . . . 1<br>NO . . . . 2<br>(»D11)	Yes. . . . . . 1<br>No 2	PROBLEM 1	PROBLEM 2		DAYS	(IF NO »D11)	PROBLEM 1	PROBLEM 2	NUMBER	(IF NO »D13)
1												
2												
3												
4												
5												
6												
7												
8												
9												
10												
11												
12												

HEALTH

D01	D12		D13	D14	D15	D16	D17	D18	D19	D20	D21	D22	D23
I<br>D<br>CODE	What kind of health provider did NAME visit? UP TO TWO PROVIDERS BY ORDER OF VISITS.		During the last 12 months, was NAME hospitalized or had an overnight stay(s) in a medical facility?	Did NAME or other members of household have to borrow money inorder to pay for hospitalization?	Did NAME or other members of household have to sell assets in order to pay for hospital-ization?	During the last 12 months, did NAME stay over-night at a traditional healer's , herbalist or faith healer's dwelling?	Did NAME or other members of household have to borrow money in order to pay for traditional healer, herbalist or faith healer?	Did NAME or other members of household have to sell assets in order to pay for traditional healer, herbalist or faith healer?	Is NAME physically handicapped in any way which limits or prevents activities or work?	Was NAME's handicap work related?	Was NAME compensated for handicap?	Was NAME compensated under any of the following?	How much did NAME receive in compensation for the handicap?
	REFERAL HOSPITAL 01<br>DISTRICT/PROVINCIAL/HOSPI 02<br>PUBLIC DISPENSARY PUBLIC 03<br>HEALTH CENTER 04<br>PRIVATE DISPENSARY/ HOSPI 05<br>PRIVATE CLINIC 06<br>TRADITIONAL HEALER 07<br>MISSIONARY HOSP./DISP 08<br>PHARMACY/CHEMIST 09<br>KIOSK 10<br>FAITH HEALER 11<br>HERBALIST 12<br>OTHER (SPECIFY) 13		YES. . . . . 1<br>NO 2	YES. . . . . 1<br>NO 2	YES. . . . . 1<br>NO 2	YES. . . . . 1<br>NO 2	YES. . . . . 1<br>NO 2	YES. . . . . 1<br>NO 2	YES. . . . . 1<br>NO 2	YES. . . . . 1<br>NO 2	YES. . . . . 1<br>NO 2	WORKMAN'S COMPENSATION.... 1<br>OWN INSURANCE COVER...... 2<br>OTHER COMPENSATION....... 3<br>EMPLOYER ARRANGEMENT.. 4	
	PROVIDER 1	PROVIDER 2	(IF NO»D16)			(IF NO »D19)			(IF NO »D25)	(IF NO »D24)	(IF NO »D24)		KSHS.
1													
2													
3													
4													
5													
6													
7													
8													
9													
10													
11													
12													

D01	D24			D25	D26	D27	D28		D29		D30	D31	D32
ID CODE	In what way(s) is NAME handicapped?			If NAME had to sweep the floor of the house, could he/she do so easily, with difficulty, or not at all?	If NAME had to walk for 2 kilometers on a flat path, could he/she do so easily, with difficulty, or not at all?	Does NAME suffer from a chronic illness?	What chronic illness does NAME suffer from? LIST UP TO 2.		How long has NAME suffered from this illness (these illnesses)?		Who diagnosed NAME's chronic illness?	Did NAME sleep under a bed net to protect against mosquitos last night?	Have the bed nets(s) ever been treated with insecticide to protect against mosquitos in the past six months?
	MISSING HAND 1<br>MISSING FOOT 2<br>LAME 3<br>BLIND 4<br>DEAF 5<br>UNABLE TO SPEAK (DUMB) 6<br>MENTALLY DISABLED 7<br>PARALYSIS 8<br>OTHER (SPECIFY) 9			EASILY 1<br>WITH DIFFICULTY 2<br>NOT AT ALL 3	EASILY 1<br>WITH DIFFICULTY 2<br>NOT AT ALL 3	YES. . . . . 1<br>NO . . . . 2<br>(»D31)	CHRONIC MALARIA/FEVER 01<br>TUBERCULOSIS 02<br>HIV/AIDS 03<br>STDs 04<br>DIABETES 05<br>ASTHMA 06<br>BILHARZIA/SCHISTOSOMIASIS 07<br>ARTHRITIS/RHEUMATISM 08<br>NERVE DISORDER 09<br>STOMACH DISORDER 10<br>SORES THAT DO NOT HEAL . . . . 11<br>CANCER. . . . . 12<br>PNEUMONIA . . . . 13<br>HIGH BLOOD PRESSURE . . . . 14<br>OTHER (SPEC.) . . . . 15<br>DO NOT KNOW (DK) 98		DO NOT KNOW 98<br>NOT STATED 99		MEDICAL WORKER (DOCTOR, CLINICAL OFFICER, NURSE) AT HOSPITAL 1<br>MEDICAL WORKER AT OTHER HEALTH FACILITY 2<br>TRADITIONAL HEALER 3<br>NON-HH MEMBER (NOT MEDICAL) 4<br>HH MEMBER 5<br>SELF 6<br>HERBALIST 7<br>FAITH HEALER 8<br>OTHERS .(specify) 9	YES. . . . . 1<br>NO . . . . 2<br>(»D33)	YES 1<br>NO 2<br>DK 8
	FIRST	SECOND	THIRD				ILLNESS 1	ILLNESS 2	YEARS	MONTHS			
1													
2													
3													
4													
5													
6													
7													
8													
9													
10													
11													
12													

FERTILITY

D01	D33	D34	D35		D36		D37		D38		D39		D40	D41
ID CODE	PUT A '1' FOR ALL FEMALES WHO ARE AGED LESS THAN 12 YRS AND MORE THAN 49YRS AND ALL MALES, OTHERWISE CODE 2.<br><br>DO NOT ADMINISTER THIS MODULE TO ALL INDIVIDUALS CODED 1.	Has NAME ever given birth to live births?	How many children have you borne alive?		How many children has NAME borne alive who usually live in the household		How many children has NAME borne alive who usually live elsewhere		How many children has NAME borne alive who have died?		When was NAME's last child born?		Sex of last child(ren) born	Is this last born child(ren) still alive?
		YES 1<br>NO 2<br>(IF NO »D42)											MALE 1<br>FEMALE 2<br>MALE TWINS 3<br>FEMALE TWINS 4<br>MULTIPLE BIRTHS 5<br>MALE - FEMALE TWINS 6	YES 1<br>NO 2<br>ONE OF THE TWINS 3<br>TWO OF THE MULTIPLE BIRTHS 4<br>ONE OF THE MULTIPLE BIRTHS 5<br>DK 8
			MALES	FEMALES	MALES	FEMALES	MALES	FEMALES	MALES	FEMALES	MONTH	YEAR		
1														
2														
3														
4														
5														
6														
7														
8														
9														
10														
11														
12														

DEATHS IN THE HOUSEHOLD

D01	D42	D43	D44		D45	D46
I D CODE	In the last 24 months has any household member died? (ask HH head or any other responsible member)<br>YES 1<br>NO 2 **(NEXT SECTION)**	Sex of person who died<br>MALE 1<br>FEMALE 2	Age of person who died<br>OVER 97 YEARS 97<br>DON'T KNOW 98<br>NOT STATED 99		Cause of Death<br>MALARIA 01<br>PNEUMONIA 02<br>AIDS 03<br>TETANUS 04<br>TUBERCULOSIS 05<br>MALNUTRITION 06<br>ANAEMIA 07<br>CHILD BIRTH/PREGNANCY 08<br>SUDDEN DEATH 09<br>ASTHMA 10<br>CANCER 11<br>URINARY OBSTRUCTION 12<br>POISONING 13<br>SUICIDE 14<br>ACCIDENT 15<br>MEASELS 16<br>OTHERS SPECIFY 17	Where did NAME die ?<br>HOME 1<br>HEALTH FACILITY 2<br>OTHERS SPECIFY 3
			YEARS	MONTHS		
1			___ ___	___ ___		
2			___ ___	___ ___		
3			___ ___	___ ___		
4			___ ___	___ ___		
5			___ ___	___ ___		
6			___ ___	___ ___		
7			___ ___	___ ___		
8			___ ___	___ ___		
9			___ ___	___ ___		
10			___ ___	___ ___		
11			___ ___	___ ___		
12			___ ___	___ ___		

SECTION D: HEALTH, FERTILITY AND HOUSEHOLD DEATHS

DEATHS IN THE HOUSEHOLD

D42	D43	D44		D45	D46
In the last 24 months has any household member died? (ask HH head or any other responsible member)	Sex of person who died	Age of person who died		Cause of Death	Where did NAME die ?
YES 1<br>NO 2 (NEXT SECTION)	MALE 1<br>FEMALE 2	OVER 97 YEARS 97<br>DON'T KNOW 98<br>NOT STATED 99		MALARIA 01<br>PNEUMONIA 02<br>AIDS 03<br>TETANUS 04<br>TUBERCULOSIS 05<br>MALNUTRITION 06<br>ANAEMIA 07<br>CHILD BIRTH/PREGNANCY 08<br>SUDDEN DEATH 09<br>ASTHMA 10<br>CANCER 11<br>URINARY OBSTRUCTION 12<br>POISONING 13<br>SUICIDE 14<br>ACCIDENT 15<br>MEASELS 16<br>OTHERS SPECIFY 17	HOME 1<br>HEALTH FACILITY 2<br>OTHERS SPECIFY 3
		YEARS	MONTHS		
		___ ___	___ ___		
		___ ___	___ ___		
		___ ___	___ ___		
		___ ___	___ ___		
		___ ___	___ ___		
		___ ___	___ ___		
		___ ___	___ ___		
		___ ___	___ ___		
		___ ___	___ ___		
		___ ___	___ ___		
		___ ___	___ ___		
		___ ___	___ ___		

SECTION E: LABOUR

[ASK ALL HOUSEHOLD MEMBERS AGED 5 YEARS AND OLDER.] IF DID NOT DO TASK, WRITE ZERO; LESS THAN 1/2 HOUR, WRITE '0.5';

E01	E02	E03	E04	E05	E06	E07	E08	E09	E10	E11	E12	E13	E14
I<br>D<br><br>C<br>O<br>D<br>E	PUT CODE '1' FOR ALL INDIVIDUALS WHO ARE AGED LESS THAN 5 YEARS OTHERWISE CODE 2<br><br>DO NOT ADMINISTER THIS MODULE TO THE INDIVIDUAL CODED 1.	What was NAME mainly doing in the past 7 days?<br><br>WORKED FOR PAY 01<br>ON LEAVE 02<br>SICK LEAVE 03<br>WORKED ON OWN/FAMILY BUSINESS 04<br>WORKED ON OWN/FAMILY AGRI.HOLDI 05<br>SEEKING WORK 06<br>(» E09)<br>DOING NOTHING 07<br>(» E09)<br>RETIRED 08<br>(» E09)<br>HOMEMAKER 09<br>(» E09)<br>FULL-TIME STUDENT 10<br>(» E14)<br>IN CAPACITATED 11<br>(» E14)<br>OTHER (SPECIFY) 12	Status in employment (Main)<br><br>PAID EMPLOYEE 1<br>WORKING EMPLOYER 2<br>OWN-ACCOUNT WORKE 3<br>UNPAID FAMILY WORKE 4<br>APPRENTICE 5<br>OTHER (SPECIFY 6	During the past 7 days, how many hours was NAME employed for a wage, salary, commission or any payment in kind?<br><br>HOURS	During the past 7 days, how many hours did NAME work on the household farm, in a field or herding livestock?<br><br>HOURS	During the past 7 days, how many hours did NAME work on any enterprise belonging to a member of household, including helping for no pay?<br><br>HOURS	REVIEW QUESTIONS E05, E06 E07: DID THE RESPONDENT WORK FOR ANY HOURS IN ANY OF THESE TASKS DURING THE LAST 7 DAYS?<br><br>YES 1<br>(»E13)<br>NO 2	Even though NAME did not do any income earning activities in the last 7 days, does NAME have a job, business, or other economic or farming activity to return to?<br><br>YES 1<br>(»E13)<br>NO 2	What is the main reason NAME was not working during the last 7 days?<br><br>SICK 01<br>RETIRED 02<br>LOOKING FOR WORK 03<br>OUT OF SEASON 04<br>RETRENCHMENT/REDUNDANCY 05<br>TEMPORARY LAY OFF 06<br>DONT NEED WORK 07<br>BUSINESS CLOSED 08<br>TOO YOUNG/TOO OLD 09<br>OTHERS 10	In the past 4 weeks has NAME taken any action to look for any kind of work or start any kind of business/income generating activity?<br><br>YES 1<br>NO 2	If NAME was offered a job, would NAME be willing to accept the job?<br><br>YES 1<br>(»E14)<br>NO 2<br>(»E14)	How many income earning activities did NAME engage in the last 7 days?<br><br>(»E15)<br>NUMBER	In the past 12 months, was NAME employed for a wage, salary, commission or any payment in kind, excluding casual labor?<br><br>YES 1<br>NO 2<br>(»E24)
1													
2													
3													
4													
5													
6													
7													
8													
9													
10													
11													
12													

E01	E15		E16		E17	E18	E19	E20
I D CODE	Describe NAME's employed occupation if worked in the last 7 days. For those who did not work during the last 7 days but worked during last 12 months give main occupation<br><br>KNOCS CODES TO BE USED FOR THIS COLUMN		Describe what <u>kind of business</u> is NAME's main occupation connected with, i.e. TYPE OF INDUSTRY?<br><br>ISIC REV 2 CODES TO BE USED FOR THIS COLUMN		Who was NAME's main <u>employer</u> for main occupation in the last 12 months<br>READ ALL RESPONSES<br><br>PRIVATE SECTOR COMPANY 01<br>INDIVIDUAL 02<br>GOVERNMENT (local auth.) 03<br>GOVERNMENT (central) 04<br>T.S.C 05<br>MAJORITY CONTROL by GOK 06<br>INTERNATIONAL ORGs. 07<br>STATE-OWNED ENTERPRISE 08<br>NGOs 09<br>OTHER (specify) 10	For how many COMPLETED months did NAME do this work during last 12 months?	For how many <u>hours per day</u> does NAME normally do this work, excluding lunch and other breaks?	How much was NAME's <u>payment for wages</u> and salary last one month (basic salary)?<br><br>IF NOT YET PAID, EXPECTED PAYMENT.
	DESCRIPTION	CODE BY SUPERVISOR<br>3 DIGIT CODES	DESCRIPTION	CODE BY SUPERVISOR<br>4 DIGIT CODES		NUMBER	HOURS	KSHS
1								
2								
3								
4								
5								
6								
7								
8								
9								
10								
11								
12								

E01	E21	E22	E23	E24	E25	E26	E27	E28	E29	E30		E31
ID CODE	How much does NAME usually receive in House Allowances that were not included in the salary NAME just reported last one month?<br><br>ESTIMATE CASH VALUE OF ANY IN-KIND PAYMENTS RECEIVED.	How much does NAME usually receive in Medical Allowances that were not included in the salary NAME just reported last one month?<br><br>ESTIMATE CASH VALUE OF ANY IN-KIND PAYMENTS RECEIVED.	How much does NAME usually receive in Other Allowances that were not included in the salary NAME just reported last one month? - ESTIMATE CASH VALUE OF ANY IN-KIND PAYMENTS RECEIVED.	At any time over past 12 months, did NAME engage in casual, part-time, labour for anyone who is not a member of your household?	For how many total days did NAME do casual labour over the past 3 months?	What was the average daily wage NAME received for the days worked at casual labour over the past 3 months?<br><br>ESTIMATE CASH VALUE OF ANY IN-KIND PAYMENTS RECEIVED.	Is NAME a member of a trade union or member of welfare association?	In which district does NAME work?	How does NAME travel to and from work most of the time?<br><br>WALK 1<br>BICYCLE 2<br>MATATU 3<br>BUS 4<br>COMMUTER TRAIN 5<br>EMPLOYER PROVIDED 6<br>PRIVATE VEHICLE 7<br>OTHER (specify) 8	How many minutes does NAME take to travel to work?		Ask Household Head or a responsible member of the household: how many domestic servants does the household employ?
	KSHS	KSHS	»E27<br>KSHS	YES 1<br>NO 2<br>»E31	DAYS	KSHS	YES 1<br>NO 2	GIVE CODE		PEAK	OFF-PEAK	NUMBER
1												
2												
3												
4												
5												
6												
7												
8												
9												
10												
11												
12												

SECTION F: CHILD HEALTH AND ANTHROPOMETRY

[FOR CHILDREN 0 TO 60 MONTHS. MOTHERS OR GUARDIANS TO ANSWER FOR CHILDREN]

F01	F02	F03	F04	F05	F06	F07	F08	F09	F10	F11	F12	F13	
I<br>D<br><br>C<br>O<br>D<br>E	PUT CODE '1' FOR ALL INDIVIDUALS WHO ARE MORE THAN 60 MONTHS IN AGE. OTHERWISE CODE 2<br><br>DO NOT ADMINISTER THIS MODULE TO INDIVIDUALS CODED 1	Where was NAME delivered?<br><br>HOSPITAL 1<br>HEALTH CENTRE 2<br>CLINIC/ DISPENSARY 3<br>MATERNITY HOME 4<br>AT HOME 5<br>OTHER 6<br>DK 9	Who assisted in birth of NAME?<br><br>DOCTOR 1<br>MIDWIFE/NURSE 2<br>TBA 3<br>TTBA 4<br>SELF 5<br>OTHER 6<br>DK 7	Has NAME ever breastfed?<br><br>YES 1<br>NO 2<br>(**»F09**)<br>DK 8<br>(**»F09**)	Is NAME still breast feeding?<br><br>YES 1<br>NO 2	For how long was NAME breastfed?<br><br>DK=98<br><br>COMPLETED<br>MONTHS	For how many months was NAME exclusively breastfed?<br><br>COMPLETED<br>MONTHS	What FIRST supplement was NAME given?<br><br>MILK OTHER THAN BREAST 1<br>COMMERCIAL INFANT FOOD/FORMULA 2<br>PORRIDGE 3<br>FORTIFIED PORRIDGE 4<br>SEMI-SOLIDS 5<br>WATER 6<br>OTHER 7<br>NA 8	Has NAME ever participated in any community nutrition programs?<br><br>YES 1<br>NO 2	Has NAME participated in the Growth Monitoring Clinic?<br><br>YES 1<br>NO 2	Has NAME had diarrhoea in the last 2 weeks?<br><br>YES 1<br>NO 2<br>(**»F16**)	What type of fluid/food was NAME given during diarrhoea?<br><br>NOTHING 1<br>COMMERCIAL INFANT/ FOOD/FORMULA/YOGURT 2<br>OTHER SEMI-SOLID FOOD 3<br>FRUITS 4<br>OTHER (*SPECIFY*) 5	 <br>NOTHING 1<br>BREASTMILK 2<br>PORRIDGE (UJI) 3<br>WATER ALONE 4<br>MILK OTHER THAN BREASTMILK 5<br>OTHER (SPECIFY) 6
												FOOD	FLUID
1													
2													
3													
4													
5													
6													
7													
8													
9													
10													
11													
12													

F01	F14		F15	F16	F17																			
I D C O D E	How much was given compared to normal times?<br>MUCH LESS 1<br>SOMEWHAT LESS 2<br>ABOUT THE SAME 3<br>MORE 4<br>DK 5		Was NAME given ORS/ORT packet solution or water, sugar and salt (*home preparation*) during diarrhea<br>YES 1<br>NO 2	Is there a vaccination card for NAME?<br>YES, SEEN 1<br>YES, NOT SEE 2 (**»F18**)<br>NO 3 (**»F18**)	Has NAME had the following vaccinations? RECORD IN DATES FROM VACCINATION CARD. (»F26)																			
					BCG						OPV (POLIO-B)						OPV1 (POLIO1)						OPV2 (POLIO2)	
	FOOD	FLUID			D	D	M	M	Y	Y	D	D	M	M	Y	Y	D	D	M	M	Y	Y	D	D
1																								
2																								
3																								
4																								
5																								
6																								
7																								
8																								
9																								
10																								
11																								
12																								

Has NAME had the following vaccinations? RECORD IN DATES FROM VACCINATION CARD. (»F26)

F01 ID CODE	OPV2 (POLIO2)				OPV3 (POLIO3)						DPT1 or DPT/HepB/Hib1						DPT2 or DPT/HepB/Hib2						DPT3 or DPT/HepB/Hib3			
	M	M	Y	Y	D	D	M	M	Y	Y	D	D	M	M	Y	Y	D	D	M	M	Y	Y	D	D	M	M
1																										
2																										
3																										
4																										
5																										
6																										
7																										
8																										
9																										
10																										
11																										
12																										

F01<br>ID CODE	MEASLES<br>Y	Y	D	D	M	M	Y	Y	F18<br>Has NAME ever received any vaccinations to prevent him/her from getting childhood diseases (including vaccinations received in a national immunization days)?<br>YES 1<br>NO 2<br>(»F26)<br>DK 3<br>(»F26)	F19<br>Has NAME ever been given a BCG vaccine against tuberculosis (injection given on the left hand and leaves a scar<br>YES 1<br>NO 2<br>DK 8	F20<br>Has NAME ever been given any vaccination drops in the mouth to protect one from getting POLIO<br>YES 1<br>NO 2<br>(»F23)<br>DK 2<br>(»F23)	F21<br>How old was NAME when the first POLIO dose was given?<br>LESS THAN SIX WEEKS 1<br>SIX WEEKS OR MORE 2<br>DK 8	F22<br>How many more times was NAME given POLIO drops since the first dose?<br>NUMBER	F23<br>Has NAME ever been given any vaccination on the thigh to prevent from getting Tetanus, Whooping cough and Diphtheria?<br>YES 1<br>NO 2<br>(»F25)<br>DK 2<br>(»F25)	F24<br>How many times was NAME given DPT doses?<br>NUMBER	F25<br>Has NAME ever been given any vaccination in the upper arm at the age of 9 months or later to prevent from measles?<br>YES 1<br>NO 2<br>DK 8	F26<br>Check whether NAME has a BCG scar on the left arm<br>YES 1<br>NO 2
1																	
2																	
3																	
4																	
5																	
6																	
7																	
8																	
9																	
10																	
11																	
12																	

F01	F27	F28	F29	F30	F31
ID CODE	PUT CODE 1 FOR ALL INDIVIDUALS WHO ARE LESS THAN 6 MONTHS IN AGE. OTHERWISE CODE 2<br><br>DO NOT ADMINISTER THIS MODULE TO INDIVIDUALS CODED 1	WEIGHT<br>IF LESS THAN 10 KG, PUT ZERO (0) IN FIRST CELL OF THIS COLUMN<br><br>KGS	HEIGHT / LENGTH MEASURED WITH CHILD STANDING OR LYING DOWN?<br><br>STANDING 1<br>LYING DOWN 2	HEIGHT<br>IF LESS THAN 100 CMS, PUT ZERO (0) IN FIRST CELL OF THIS COLUMN<br><br>CMS	IF NAME NOT MEASURED, REASON WHY?<br><br>NOT HOME DURING SURVEY PERIOD 1<br>TOO ILL 2<br>UNWILLING 3<br>OTHER 4
1					
2					
3					
4					
5					
6					
7					
8					
9					
10					
11					
12					

SECTION G: HOUSING

[ASK OF HOUSEHOLD HEAD OR KNOWLEDGEABLE HOUSEHOLD MEMBER]

G01	G02	G03	G04	G05	G06	G07	G08	G08A	G09	
What is HH tenure status of main residence?<br>OWNER OCCUPIED 1<br>OWNER OCCUPIED NOMADS 2<br>EMPLOYER PROVIDED SUBSIDIZED 3 (»G06)<br>EMPLOYER PROVIDED FREE 4 (»G08)<br>RENTED (»G06) 5<br>FREE (»G08) 6	IF OWNER-OCCUPIED, how did HH acquire this dwelling?<br>PURCHASED CASH 1 (»G04)<br>PURCHASED LOAN 2<br>CONSTRUCTED CASH 3 (»G04)<br>CONSTRUCTED LOAN 4<br>PURCHASED CASH & LOAN 5<br>CONSTRUCTED CASH & LOAN 6<br>INHERITED 7<br>GIFT 8 (»G04)<br>BARTERED 9 (»G04)	How much per month is HH spending to service the loans on housing?<br>KSHS	How many years ago was this house built?<br>(How old is it?)<br>D K 98<br>YEARS	If you sold this dwelling today, how much would it sell for?<br>KSHS	From whom do HH rent ?<br>GOVERNMENT 1<br>LOCAL AUTHORITY 2<br>PARASTATAL 3<br>COMPANY 4<br>INDIVIDUAL 5<br>NA 7 (»G08)<br>OTHER (SPECIFY) 9	How much does HH pay to rent this dwelling?<br>(KSHS PER MONTH)<br>KSHS	Estimate the rent HH could receive/pay if rented this dwelling or one exactly like it to another person?<br>(KSHS PER MONTH)<br>KSHS	How many dwelling units does this household	How many habitable rooms does this HH occupy?<br>(DO NOT COUNT BATHROOMS, TOILETS, STOREROOMS, OR GARAGE)<br>MAIN HOUSE<br>NUMBER OF ROOMS	OTHER DWELLINGS<br>NUMBER OF ROOMS

G10		G11	G12	G13	G14	G15	G16	G17	G18
What is the total floor area of all the rooms in Sq. M.?<br>MAIN HOUSE<br>FLOOR AREA OF ROOMS	OTHER DWELLINGS<br>FLOOR AREA OF ROOMS	WHAT TYPE OF DWELLING DOES THE HOUSEHOLD LIVE IN?<br>HOUSE/BUNGALOW 1<br>FLAT 2<br>MAISONNETT 3<br>SWAHILI 4<br>SHANTY 5<br>MANYATTA/TRADITIONAL HSE 6<br>OTHER (SPECIFY) 7	THE WALLS OF THE MAIN DWELLING IS PREDOMINANTLY MADE OF WHAT<br>STONE 01<br>BRICK/BLOCK 02<br>MUD/WOOD 03<br>MUD/CEMENT 04<br>WOOD ONLY 05<br>CORRUGATED IRON SHEI 06<br>GRASS/STRAW 07<br>TIN 08<br>OTHER (SPECIFY) 09	THE ROOF OF THE MAIN DWELLING IS PREDOMINANTLY MADE OF WHAT MATERIAL?<br>CORRUGATED IRON SHEET 1<br>TILES 2<br>CONCRETE 3<br>ASBESTOS SHEETS 4<br>GRASS 5<br>MAKUTI 6<br>TIN 7<br>OTHER (SPECIFY) 8	THE FLOOR OF THE MAIN DWELLING IS PREDOMINANTLY MADE OF WHAT MATERIAL?<br>CEMENT 1<br>TILES 2<br>WOOD 3<br>EARTH 4<br>OTHER (SPECIFY) 5	What is the location of the main cooking area/kitchen?<br>OUTDOOR 1<br>ENCLOSED DETACHE 2<br>ENCLOSED ATTACHE 3<br>INDOOR WITHOUT PARTITION 4<br>INDOOR WITH PARTITION 5<br>OTHER (SPECIFY) 6	What is the main primary type of appliance used for cooking?<br>TRADITIONAL STONE FIRE 1<br>IMPROVED TRADITIONAL STONE FIRE 2<br>ORDINARY JIKO 3<br>IMPROVED JIKO 4<br>KEROSENE STOVI 5<br>GAS COOKER 6<br>ELECTRIC COOKE 7<br>OTHER (SPECIFY 8	Does the household's kitchen have a chimney?<br>YES 1<br>NO 2	Is the toilet facility located within the dwelling unit?<br>YES 1<br>NO 2

SECTION H: WATER, SANITATION AND ENERGY USE

[ASK OF HOUSEHOLD HEAD OR KNOWLEDGEABLE HOUSEHOLD MEMBER]

WATER																			
H01<br>What is HH main source water over the past month?<br><br>PIPED INTO DWELLING 01<br>PIPED INTO PLOT/YARD 02<br>PUBLIC TAP 03<br>TUBEWELL/BOREHOLE WITH PUMP 04<br>PROTECTED DUG WELL 05<br>PROTECTED SPRING 06<br>RAIN WATER COLLECTION 07<br>UNPROTECTED DUG WELL/SPRINGS 08<br>RIVER/PONDS/STREAMS 09<br>TANKERS-TRUCK/VENDOR 10<br>BOTTLED WATER 11<br>OTHER (SPECIFY) 12					H02<br>How long does it take (MINUTES) to walk (ONE WAY) to this main water source from dwelling?<br><br>IF IN DWELLING, ENTER 0<br><br>GIVE TIME IN MINUTES					H03<br>Typically when you get to this source, how much time (MINUTES) does one have to wait in a queue to collect the water?<br><br>IF IN<br><br>IF IN DWELLING GIVE 0<br><br>MINUTES	H04<br>Do you use this source for your drinking water<br><br>ALL YEAR 1<br>(»H07)<br>ONLY DRY SEASON 2<br>ONLY RAINY SEASON 3	H05<br>In the other season, what is your main source of drinking water?<br><br>USE CODES FOR H01<br><br>CODE	H07<br>How do you mainly store water at home?<br><br>DO NOT 1<br>BUCKET/JERRY CAN 2<br>DRUMS CONTAINER 3<br>WATER TANK 4<br>OTHER(SPECIFY) 5	H08<br>What was the total cost of water for your household last month?<br><br>IF NONE, ENTER ZERO (»H10)<br><br>KSHS				
DRINKING	BATHING	COOKING	LIVESTOCK	OTHER	DRINKING	BATHING	COOKING	LIVESTOCK	OTHER					DRINKING	BATHING	COOKING	WASHING	TOTAL

WATER			SANITATION						ENERGY			
H09<br>Approximately how many jerry cans (20 litres) of water does this total payment cover per month?<br><br>NUMBER	H10<br>How many 20 litres jerry cans does the HH on average consume per day?<br><br>NUMBER	H11<br>How much does one jerrycan of water costs on average?<br><br>KSHS	H12<br>Does all HH members use a common (same) type of toilet?<br><br>YES 1<br>NO 2	H13<br>What is the main toilet facilities for this household?<br><br>FLUSH TOILET (»H15) 1<br>VIP LATRINE 2<br>UNCOVERED PIT LATRINE 3<br>COVERED PIT LATRINE 4<br>BUCKET . . .<br>NONE (»H17) 5<br>OTHER (SPECIFY) 6	H14<br>How deep is the latrine?<br><br>SHALLOW...1<br>DEEP 2	H15<br>Is this toilet facility for the use of:<br><br>READ OPTIONS GIVEN BELOW<br><br>HH MEMBERS ONLY 1<br>2 HH 2<br>3 HH 3<br>4 OR MORE 4	H16<br>Who built/paid for building or provided this toilet?<br><br>HOUSEHOLD BUILT 1<br>LANDLORD 2<br>NEIGHBOR 3<br>COMMUNITY PROGRAM 4<br>LOCAL AUTHORITY 5<br>OTHER (SPECI 6	H17<br>How does the household dispose of its garbage?<br><br>COLLECTED BY LOCAL AUTHORITY 1<br>COLLECTED BY PRIVATE FIRM 2<br>GARBAGE PIT 3<br>BURNING 4<br>PUBLIC GARBAGE HEAP 5<br>FARM/GARDEN 6<br>NEIGHBOURHOOD COMMUNITY GROUP 7<br>OTHER 8	H18<br>What is Household's main sources of cooking fuel?<br><br>COLLECTED FIREWOOD 01<br>PURCHASED FIREWOOD 02<br>GRASS 03<br>PARAFFIN 04<br>ELECTRICITY 05<br>GAS/LPG 06<br>CHARCOAL 07<br>BIOMASS RESIDUE 08<br>BIOGAS 09<br>OTHER 10		H18A<br>What is HH TWO main sources of lighting fuel?<br><br>(IF SOURCE NOT ELECTRICITY OR SOLAR »H26)<br><br>COLLECTED FIREWOOD 01<br>PURCHASED FIREWOOD 02<br>GRASS 03<br>PARAFFIN 04<br>ELECTRICITY 05<br>SOLAR 06<br>GAS 07<br>DRY CELL (TORCH) 08<br>CANDLES 09<br>BIOGAS 10<br>OTHER 11	
									Main	2nd	Main	2nd

ENERGY												
H19	H20	H21	H22	H23	H24	H25	H26	H27	H28	H29	H30	H31
Does HH have installed solar panels in the dwelling?<br>YES 1<br>NO 2<br>**>>H21**	What is the size of solar panels in watts?<br>DK 998<br>WATTS	Does HH get electricity from KPLC?<br>YES 1<br>NO 2	Does HH get any electricity from generator?<br>OWN GENERATOR 1<br>NEIGHBOR GENERATOR 2<br>NO 3<br>OTHER (specif 4	Does HH get electricity from car or motorcycle battery?<br>CAR 1<br>MOTOR CYCLE 2<br>BOTH 3<br>NO 4	What is HH main source of electricity?<br>KPLC 1<br>COMMUNITY GENERATOR 2<br>SOLAR PANELS 3<br>GENERATOR, OWN 4<br>CAR BATTERY 5<br>MOTORCYCLE BATTERY 6<br>OTHER (SPECIFY) 7	What was the total cost for electricity in the household over the last month?<br>IF THE HOUSEHOLD RECEIVED AN ACTUAL BILL, REFER TO THE LAST BILL RECEIVED. INCLUDE OTHER PAYMENTS/COST OF ELECTRICITY WHICH DO NOT COME ON A WRITTEN BILL.<br>KSHS	What is the total cost of lighting last month (excluding electricity)?<br>KSHS	Does HH usually have any electricity working in the dwelling?<br>YES 1<br>**(»H29)**<br>NO 2	Although you do not have electricity here, are there HH connected within 100m from the dwelling?<br>YES 1<br>NO 2	Did household use small torch batteries (dry cells) in the last one month?<br>YES 1<br>NO 2<br>**(»H32)**	How many small torch batteries(dry cells) did household use in the past one<br>NUMBER	What is the value of these batteries (dry cells) used in the past one month?<br>KSHS

SECTION H: WATER, SANITATION AND ENERGY USE

[ASK OF HOUSEHOLD HEAD OR KNOWLEDGEABLE HOUSEHOLD MEMBER]

ENERGY

H32	ENERGY SOURCES.<br><br>COMPLETE H32- H40 FOR EACH ENERGY SOURCE BEFORE PROCEEDING TO THE NEXT ENERGY SOURCE.	H33<br>In the last 12 months, has your household used [ITEM]?<br><br>YES 1<br>NO 2<br>(**»NEXT ITEM**)	H34<br>In the last month, has your household used [ITEM]?<br><br>YES 1<br>NO 2<br>(**»NEXT ITEM**)	H35<br>What was the main purpose of this use?<br><br>BOILING 1<br>HEATING 2<br>COOKING 3<br>LIGHTING 4<br>OTHER 5	H36<br>What unit(s) of measure and quantity do you use for [ITEM]?<br><br>BUNCH 1<br>BUNDLE 2<br>HEAP 3<br>LOG 4<br>PIECE 5<br>SACK 6<br>DEBE 7<br>GOROGORO 8<br>TIN 9<br>OTHERS SPECIFY 10<br><br>Unit	 <br><br><br><br><br><br><br><br><br><br><br><br><br><br>Quantity	H37<br>What is the estimated weight of a typical unit of [ITEM]?<br><br>ASK TO SEE A TYPICAL STACK/ BUNDLE/ PIECE TO ESTIMATE WEIGHT<br><br>KGS	H38<br>During the last month, how many units of [ITEM] did you use?<br><br>FOR LPG (GAS), USE 1 DECIMAL AS NEEDED<br><br>NUMBER	 3 KG CYLINDER 1<br>6 KG CYLINDER 2<br>12.5 KG CYLINDER 3<br>13 KG CYLINDER 4<br>15 KG CYLINDER 5<br>OTHER (SPECIFY) 6<br><br>UNIT	H39<br>What was the total cost of these units that you used during the last month?<br><br>KSHS	H40<br>How long would one take to the nearest source one-way distance travelled to get [ITEM] in MINUTES?<br><br>MINUTES	H41<br>How many minutes per week was used to get [ITEM] by members of your household?<br><br>MEN	 WOMEN	 CHILDREN
1	PURCHASED FIREWOOD													
2	COLLECTED FIREWOOD													
3	ANIMAL WASTE (BIOMASS RESIDUE)													
4	STRAW OR STALK (BIOMASS RESIDUE)													
5	CHARCAOL													
6	KEROSENE/PARAFIN									LITRES					
7	GAS/LPG													
8	ELECTRICITY									WATTS					

SECTION I: CONSUMPTION OF FOOD OVER PAST ONE WEEK

PROMPT FOR EACH ITEM ON THE LIST.

Over the past one week (7 days), did household acquire / purchased / consumed any [. . .]?	I01	I02	I03 How much was purchased?			I04 How much of the purchased was consumed?	I05 How much was consumed from own-production?		I05A How much was consumed from own stock?		I06 How much was consumed from gifts and other sources?		I07 How much in total did your household consume in the past week?	
	YES 1<br>NO 2<br>(»NEXT ITEM)	ITEM CODE	QUANTITY	UNIT CODES AT RIGHT	KSHS	QUANTITY	QUANTITY	UNIT CODES AT RIGHT	QUANTITY	UNIT CODES AT RIGHT	QUANTITY	UNIT CODES AT RIGHT	QUANTITY	UNIT CODES AT RIGHT
**CEREALS**		100												
Rice Grade 2		101												
Rice- Grade 1 - Pishori/Basmati		102												
Maize Grain - Loose		103												
Green maize		104												
Maize Flour - Loose		105												
Maize Flour - sifted		106												
Wheat grain		107												
Wheat Flour		108												
Millet grain - wimbi		109												
Millet Flour - Wimbi		110												
Sorghum grain		111												
Sorghum flour		112												
Other millet grain/flour		113												
Barley and other cereals		114												
COST OF MILLING		115												
Bread		116												
Cakes		117												
Biscuits ...		118												
Breakfast cereal/oats		119												
Wheat buns /Scones		120												
Pasta (spaghetti/macaroni)		121												

KILOGRAMS	01
GRAMS	02
LITRE	03
MILLILITRE	04
5 KG. BAG	05
25 KG. BAG	06
50 KG. BAG	07
90 KG. BAG	08
DEBE	09
TABLE SPOON	10
BUNCH	11
PIECE/NUMBER	12
HEAP	13
HANDFUL	14
PAKAACHA	15
GOROGORO	16
1/4 KG TIN	17
1/2 KG TIN	18
1 KG TIN	19
BOWL	20
CUP	21
GLASS	22

SECTION I: CONSUMPTION OF FOOD OVER PAST ONE WEEK

PROMPT FOR EACH ITEM ON THE LIST.

	I01	I02	I03			I04	I05		I05A		I06		I07			
Over the past one week (7 days), did household acquire / purchased / consumed any [. . .]?	YES 1<br>NO 2		How much was purchased?			How much of the purchased was consumed?	How much was consumed from own-production?		How much was consumed from own stock?		How much was consumed from gifts and other sources?		How much in total did your household consume in the past week?			
	(»NEXT ITEM)	ITEM CODE	QUANTITY	UNIT CODES AT RIGHT	KSHS	QUANTITY	QUANTITY	UNIT CODES AT RIGHT	QUANTITY	UNIT CODES AT RIGHT	QUANTITY	UNIT CODES AT RIGHT	QUANTITY	UNIT CODES AT RIGHT		
**Roots and tubers**		200													KILOGRAMS	01
Potatoes (Irish)		201													GRAMS	02
Sweet potato		202													LITRE	03
Arrow roots		203													MILLILITRE	04
Cassava		204													5 KG. BAG	05
Cassava flour		205													25 KG. BAG	06
Yams		206													50 KG. BAG	07
Crisps		207													90 KG. BAG	08
Cooking banana		208													DEBE	09
**Pulses**		300													TABLE SPOON	10
Beans		301													BUNCH	11
Grams		302													PIECE/NUMBER	12
Black grams (Njahi)		303													HEAP	13
Peas		304													HANDFUL	14
Groundnut		305													PAKAACHA	15
Cowpea		306													GOROGORO	16
Other pulses (specify)		307													1/4 KG TIN	17
**Vegetables**		400													1/2 KG TIN	18
onion / Leeks		401													1 KG TIN	19
Cabbages		402													BOWL	20
Carrots		403													CUP	21
Tomatoes		404													GLASS	22
Spinach		405													OTHER (SPECIFY)	23

SECTION I: CONSUMPTION OF FOOD OVER PAST ONE WEEK

PROMPT FOR EACH ITEM ON THE LIST.

Over the past one week (7 days), did household acquire / purchased / consumed any [. . .]?	I01	I02	I03 How much was purchased?			I04 How much of the purchased was consumed?	I05 How much was consumed from own-production?		I05A How much was consumed from own stock?		I06 How much was consumed from gifts and other sources?		I07 How much in total did your household consume in the past week?	
	YES 1<br>NO 2<br>(»NEXT ITEM)	ITEM CODE	QUANTITY	UNIT CODES AT RIGHT	KSHS	QUANTITY	QUANTITY	UNIT CODES AT RIGHT	QUANTITY	UNIT CODES AT RIGHT	QUANTITY	UNIT CODES AT RIGHT	QUANTITY	UNIT CODES AT RIGHT
Kale-Sukuma wiki		406												
Capsicums (Pilipili hoho)		407												
Cucumber		408												
French beans		409												
Lettuce		410												
Courgette		411												
Celery		412												
mushrooms		413												
Cauliflower		414												
Aubergines-Egg plant (Biringanya)		415												
Pumpkins		416												
Okra		417												
Coriander leaves (Dania)		418												
Other vegetables (specify: ________)		419												
**Meat**		500												
Beef - with bones		501												
Beef - without bones		502												
Minced meat		503												
Pork		504												
Mutton/Goat meat		505												
Chicken		506												
Camel meat		507												
Other Meats (specify)		508												

KILOGRAMS	01
GRAMS	02
LITRE	03
MILLILITRE	04
5 KG. BAG	05
25 KG. BAG	06
50 KG. BAG	07
90 KG. BAG	08
DEBE	09
TABLE SPOON	10
BUNCH	11
PIECE/NUMBER	12
HEAP	13
HANDFUL	14
PAKAACHA	15
GOROGORO	16
1/4 KG TIN	17
1/2 KG TIN	18
1 KG TIN	19
BOWL	20
CUP	21
GLASS	22
OTHER (SPECIFY)	23

SECTION I: CONSUMPTION OF FOOD OVER PAST ONE WEEK

PROMPT FOR EACH ITEM ON THE LIST.

Over the past one week (7 days), did household acquire / purchased / consumed any [. . .]?	I01 YES 1 NO 2	I02	I03 How much was purchased?			I04 How much of the purchased was consumed?	I05 How much was consumed from own-production?		I05A How much was consumed from own stock?		I06 How much was consumed from gifts and other sources?		I07 How much in total did your household consume in the past week?	
	(»NEXT ITEM)	ITEM CODE	QUANTITY	UNIT CODES AT RIGHT	KSHS	QUANTITY	QUANTITY	UNIT CODES AT RIGHT	QUANTITY	UNIT CODES AT RIGHT	QUANTITY	UNIT CODES AT RIGHT	QUANTITY	UNIT CODES AT RIGHT
**Other Animal products**		600												
Offal's (liver, kidney, etc)-Matumbo		601												
Sausages		602												
Bacon		603												
Ham/Salami		604												
Corned beef		605												
**Fish**		700												
Fresh fish		701												
Frozen Fish Filets		702												
Dried/smoked fish		703												
Prawns /Other sea Foods		704												
**Dairy products and eggs**		800												
Milk - fresh unpacketed		801												
milk - fresh packeted		802												
milk - fresh flavoured packeted		803												
UHT- fresh flavoured Milk		804												
Milk - condensed/powder		805												
Baby milk - tinned		806												
Milk Sour - Mala		807												
Yogurt (clotted milk)		808												
Fresh cream		809												
Cheese		810												
Eggs		811												

KILOGRAMS	01
GRAMS	02
LITRE	03
MILLILITRE	04
5 KG. BAG	05
25 KG. BAG	06
50 KG. BAG	07
90 KG. BAG	08
DEBE	09
TABLE SPOON	10
BUNCH	11
PIECE/NUMBER	12
HEAP	13
HANDFUL	14
PAKAACHA	15
GOROGORO	16
1/4 KG TIN	17
1/2 KG TIN	18
1 KG TIN	19
BOWL	20
CUP	21
GLASS	22
OTHER (SPECIFY)	23

SECTION I: CONSUMPTION OF FOOD OVER PAST ONE WEEK

PROMPT FOR EACH ITEM ON THE LIST.

Over the past one week (7 days), did household acquire / purchased / consumed any [. . .]?	I01	I02	I03 How much was purchased?			I04 How much of the purchased was consumed?	I05 How much was consumed from own-production?		I05A How much was consumed from own stock?		I06 How much was consumed from gifts and other sources?		I07 How much in total did your household consume in the past week?	
	YES 1<br>NO 2<br>(»NEXT ITEM)	ITEM CODE	QUANTITY	UNIT CODES AT RIGHT	KSHS	QUANTITY	QUANTITY	UNIT CODES AT RIGHT	QUANTITY	UNIT CODES AT RIGHT	QUANTITY	UNIT CODES AT RIGHT	QUANTITY	UNIT CODES AT RIGHT
**Oils and Fats**		900												
Butter		901												
Ghee from milk		902												
Margarine		903												
Cooking Fat		904												
Cooking oil		905												
Lard (from butcheries)		906												
Peanut butter		907												
**Fruits**		1000												
Banana - ripe		1001												
Oranges		1002												
Pawpaws		1003												
Avocado		1004												
Mangoes		1005												
Pineapples		1006												
Passion fruits		1007												
Pears		1008												
Peaches		1009												
Plums		1010												
Apples		1011												
Lemons		1012												
Grape fruit		1013												
Strawberries		1014												

KILOGRAMS	01
GRAMS	02
LITRE	03
MILLILITRE	04
5 KG. BAG	05
25 KG. BAG	06
50 KG. BAG	07
90 KG. BAG	08
DEBE	09
TABLE SPOON	10
BUNCH	11
PIECE/NUMBER	12
HEAP	13
HANDFUL	14
PAKAACHA	15
GOROGORO	16
1/4 KG TIN	17
1/2 KG TIN	18
1 KG TIN	19
BOWL	20
CUP	21
GLASS	22
OTHER (SPECIFY)	23

SECTION I: CONSUMPTION OF FOOD OVER PAST ONE WEEK

PROMPT FOR EACH ITEM ON THE LIST.

Over the past one week (7 days), did household acquire / purchased / consumed any [. . .]?	I01	I02	I03 How much was purchased?			I04 How much of the purchased was consumed?	I05 How much was consumed from own-production?		I05A How much was consumed from own stock?		I06 How much was consumed from gifts and other sources?		I07 How much in total did your household consume in the past week?	
	YES 1<br>NO 2<br>(»NEXT ITEM)	ITEM CODE	QUANTITY	UNIT CODES AT RIGHT	KSHS	QUANTITY	QUANTITY	UNIT CODES AT RIGHT	QUANTITY	UNIT CODES AT RIGHT	QUANTITY	UNIT CODES AT RIGHT	QUANTITY	UNIT CODES AT RIGHT
Melons		1015												
Grapes		1016												
Coconut		1017												
Strawberries		1018												
Other berries		1019												
Sugar cane		1024												
Squashes		1020												
Health Drink		1021												
Preserved fruit, juice		1022												
Other fruits (specify)		1023												
**Sugar**		1100												
Sugar		1101												
Jaggery (Nguru)		1102												
Sugar-icing		1103												
Other sugar (confectionary, etc)		1104												
**Jam, Honey, Sweets and candies**		1200												
Jam		1201												
Marmalade		1202												
Honey		1203												
Chocolate		1204												
Sweets		1205												
Chewing gum		1206												

KILOGRAMS	01
GRAMS	02
LITRE	03
MILLILITRE	04
5 KG. BAG	05
25 KG. BAG	06
50 KG. BAG	07
90 KG. BAG	08
DEBE	09
TABLE SPOON	10
BUNCH	11
PIECE/NUMBER	12
HEAP	13
HANDFUL	14
PAKAACHA	15
GOROGORO	16
1/4 KG TIN	17
1/2 KG TIN	18
1 KG TIN	19
BOWL	20
CUP	21
GLASS	22
OTHER (SPECIFY)	23

SECTION I: CONSUMPTION OF FOOD OVER PAST ONE WEEK

PROMPT FOR EACH ITEM ON THE LIST.

	I01	I02	I03			I04	I05		I05A		I06		I07	
Over the past one week (7 days), did household acquire / purchased / consumed any [. . .]?	YES 1<br>NO 2<br>(»NEXT ITEM)		How much was purchased?			How much of the purchased was consumed?	How much was consumed from own-production?		How much was consumed from own stock?		How much was consumed from gifts and other sources?		How much in total did your household consume in the past week?	
		ITEM CODE	QUANTITY	UNIT CODES AT RIGHT	KSHS	QUANTITY	QUANTITY	UNIT CODES AT RIGHT	QUANTITY	UNIT CODES AT RIGHT	QUANTITY	UNIT CODES AT RIGHT	QUANTITY	UNIT CODES AT RIGHT
**Beverages for drinking**		1300												
Drinking chocolate/other cocoa preparations		1301												
Soya drink		1302												
Coffee - instant		1303												
Grounded coffee		1304												
Tea Leaves		1305												
Tea Bags		1306												
**Non-alcoholic beverages**		1400												
Mineral water		1401												
sodas		1402												
**Alcoholic beverages**		1500												
Spirits		1501												
Wine		1502												
Beer		1503												
Traditional brew - muratina, buzaa ,chang'aa		1504												
Cider		1505												
**Tobacco**		1600												
Cigarettes		1601												
Tobacco -processed		1602												
Tobacco -raw		1603												
Cigars		1604												
Snuff		1605												
Miraa (Khat)		1606												

KILOGRAMS	01
GRAMS	02
LITRE	03
MILLILITRE	04
5 KG. BAG	05
25 KG. BAG	06
50 KG. BAG	07
90 KG. BAG	08
DEBE	09
TABLE SPOON	10
BUNCH	11
PIECE/NUMBER	12
HEAP	13
HANDFUL	14
PAKAACHA	15
GOROGORO	16
1/4 KG TIN	17
1/2 KG TIN	18
1 KG TIN	19
BOWL	20
CUP	21
GLASS	22
OTHER (SPECIFY)	23

SECTION I: CONSUMPTION OF FOOD OVER PAST ONE WEEK

PROMPT FOR EACH ITEM ON THE LIST.

Over the past one week (7 days), did household acquire / purchased / consumed any [. . .]?	I01<br>YES 1<br>NO 2<br>(»NEXT ITEM)	I02<br>ITEM CODE	I03 How much was purchased?<br>QUANTITY	UNIT CODES AT RIGHT	KSHS	I04 How much of the purchased was consumed?<br>QUANTITY	I05 How much was consumed from own-production?<br>QUANTITY	UNIT CODES AT RIGHT	I05A How much was consumed from own stock?<br>QUANTITY	UNIT CODES AT RIGHT	I06 How much was consumed from gifts and other sources?<br>QUANTITY	UNIT CODES AT RIGHT	I07 How much in total did your household consume in the past week?<br>QUANTITY	UNIT CODES AT RIGHT
**Spices & Miscellaneous**		1700												
Salt		1701												
Tomato sauce		1702												
Chilli sauce		1703												
Other spices (Specify)		1704												
Baking Powder /Bicarbonate		1705												
Yeast		1706												
Mustard		1707												
Tomato Sauce		1708												
Vinegar		1709												
Pickles		1710												
**Tinned products**		**1800**												
Tinned Beans		1801												
Tinned pulses		1802												
Tinned /packeted soups (vegetables)		1803												
Tinned /packeted soups (meat)		1804												
Tinned fish		1805												
Baby food- Cereals (tinned)		1806												
**Cooked/Prepared Foods from Vendors**		1900												
Food from Vendors		1901												
Cafe and take-aways: food		1902												
Kiosks: food		1903												
Restaurants and hotels: food		1904												

KILOGRAMS	01
GRAMS	02
LITRE	03
MILLILITRE	04
5 KG. BAG	05
25 KG. BAG	06
50 KG. BAG	07
90 KG. BAG	08
DEBE	09
TABLE SPOON	10
BUNCH	11
PIECE/NUMBER	12
HEAP	13
HANDFUL	14
PAKAACHA	15
GOROGORO	16
1/4 KG TIN	17
1/2 KG TIN	18
1 KG TIN	19
BOWL	20
CUP	21
GLASS	22
OTHER (SPECIFY)	23

SECTION J: REGULAR NON FOOD ITEMS EXPENDITURES - PAST ONE MONTH

PROMPT FOR EACH ITEM ON THE LIST.

Over the past one month, did any member of the household acquire/purchase /consume any [. . .]?	J01 YES 1 NO 2 (»NEXT ITEM)	J02 ITEM CODE	J03 How much was purchased? QUANTITY	UNIT CODES IN SECTION I	KSHS	J04 How much was acquired/obtained from other sources? QUANTITY	UNIT CODES IN SECTION I	KSHS
**Household Operation**		2000						
Household Soap/bar soap		2001						
Detergents		2002						
Dish washing paste/liquid		2003						
insecticide		2004						
Disinfectant		2005						
Air freshener		2006						
Floor polish		2007						
Broom		2008						
Mop/Duster		2009						
Shoe polish /Cream		2010						
Match box		2011						
Candles		2012						
Laundry (clothes)		2013						
Laundry (others specify)		2014						
**Personal Care and Effects**		3000						
Hair cut (men)		3001						
Sanitary pads		3002						
Cotton wool		3003						
Baby oil		3004						
Baby powder		3005						
Hair oil		3006						
Hair oil		3007						

PROMPT FOR EACH ITEM ON THE LIST.

Over the past one month, did any member of the household acquire / purchase / consume any [. . .]?	J01 YES 1 NO 2 (»NEXT ITEM)	J02 ITEM CODE	J03 How much was purchased? QUANTITY	UNIT CODES IN SECTION I	KSHS	J04 How much was acquired/obtained from other sources? QUANTITY	UNIT CODES IN SECTION I	KSHS
**Personal Care and Effects**		3000						
Perfume		3008						
Other toiletries specify)		3009						
Other cosmetics specify)		3010						
Massage		3011						
Hair dressing (women)		3012						
massage (ladies)		3013						
Razor/Blade		3014						
Combs		3015						
Toothbrush		3016						
Toilet soap		3017						
Toilet paper		3018						
Toothpaste		3019						
After Shave lotion		3020						
Body lotion		3021						
Hair cream		3022						
Shampoo/conditioner		3023						
Deodorant		3024						
Tissue paper/Handkerchiefs		3025						
Petroleum jelly		3026						
Nail polish		3027						
Lipstick		3028						
Eye make-ups		3029						

SECTION J: REGULAR NON FOOD ITEMS EXPENDITURES - PAST ONE MONTH

PROMPT FOR EACH ITEM ON THE LIST.

	J01	J02	J03			J04		
Over the past one month, did any member of the household acquire/purchase /consume any [. . .]?	YES 1<br>NO 2		How much was purchased?			How much was acquired/obtained from other sources?		
	(»NEXT ITEM)	ITEM CODE	QUANTITY	UNIT CODES IN SECTION I	KSHS	QUANTITY	UNIT CODES IN SECTION I	KSHS
**Medical Care**		3100						
Medicines - Antiworms		3101						
Liver salts and other anti-acids		3102						
Cold tablets/cough syrup		3103						
Herbal medicine		3104						
Balms (healing ointments)		3105						
Vaccines		3106						
Contraceptives		3107						
Adhesive bandage		3108						
Syringes		3109						
Multivitamin/other medicine		3110						
Fever/Pain killers e.g. paracetemol		3111						
Medicine anti-malaria		3112						
Cod/halibut liver oil		3113						
**Transport and Communication**		3200						
Petrol		3201						
Diesel		3202						
Ferry/road tolls		3203						
Taxi Fare		3204						
Parking charges		3205						
City bus fares		3206						
Country bus fare		3207						
Matatu fares		3208						

PROMPT FOR EACH ITEM ON THE LIST.

	J01	J02	J03			J04		
Over the past one month, did any member of the household acquire / purchase / consume any [. . .]?	YES 1<br>NO 2		How much was purchased?			How much was acquired/obtained from other sources?		
	(»NEXT ITEM)	ITEM CODE	QUANTITY	UNIT CODES IN SECTION I	KSHS	QUANTITY	UNIT CODES IN SECTION I	KSHS
**Transport and Communication**		3200						
Boda boda fares		3209						
Train fares		3210						
Local flights		3211						
Local calls		3212						
Trunk calls		3213						
Cell phone airtime		3214						
Internet costs		3215						
Refuse collection		3216						
**Fuel and Power**		3300						
Electricity		3301						
GAS/LPG		3302						
Kerosene/Paraffin		3303						
Firewood		3304						
Charcoal		3305						
Other cooking fuels		3306						
Water		3307						

SECTION K: NON FOOD ITEMS EXPENDITURES - PAST ONE MONTH

PROMPT FOR EACH ITEM ON THE LIST.

Over the past one month, did any member of the household acquire, purchase or consumed any [...]?	K0 YES 1 NO 2 (»NEXT ITEM)	K02 ITEM CODE	K03 How much was purchased/paid for? QUANTITY	UNIT CODES IN SECTION I	KSHS	K04 How much was acquired/obtained from other sources? QUANTITY	UNIT CODES IN SECTION I	KSHS
**Domestic Services**		3300						
Domestic workers		3301						
**Upkeep of Housing Unit**		3400						
Maintenance and repair		3401						
Additions/improvement		3402						
Additions/improvement (owner occupiers)		3403						
**Personal Goods**		3500						
Jewelers		3501						
Belts		3502						
Watches		3503						
Purses/handbags (ladies)		3504						
wallets (men)		3505						
Suitcase/brief cases		3506						
Travel bags		3507						
Umbrellas		3508						
Batteries (dry cells)		3509						
Personal Torches		3510						
Clock		3511						
Smoke Pipes		3512						
Lighters		3513						
**Recreation and Entertainment**		3600						
Video Cassette hire		3601						
Films purchase/developing		3602						

PROMPT FOR EACH ITEM ON THE LIST.

Over the past one month, did any member of the household acquire, purchase or consumed any [...]?	K01 YES 1 NO 2 (»NEXT ITEM)	K02 ITEM CODE	K03 How much was purchased/paid for? QUANTITY	CODES IN SECTION I	KSHS	K04 How much was acquired/obtained from other sources? QUANTITY	CODES IN SECTION I	KSHS
**Recreation and Entertainment**		3600						
cinema entry fees		3603						
Stadium entrance fees		3604						
National /Game park entry fees		3605						
Traditional dances		3606						
Disco/night club entry fees		3607						
Gambling/lottery tickets		3608						
Books		3609						
Newspapers		3610						
Magazines		3611						
Pencils/pens/ink		3612						
Envelopes-		3613						
Writing pad/Exercise books		3614						
Photocopying		3615						
**Medical Care**		3700						
Doctor's service -General Practitioner		3701						
Traditional Doctor's service		3702						
Specialist		3703						
Dental services		3704						
Nurses		3705						
Spectacles		3706						
Optician's services		3707						
Physiotherapy (out-patients)		3708						

PROMPT FOR EACH ITEM ON THE LIST.

Over the past one month, did any member of the household acquire, purchase or consumed any [...]?	K0<br>YES 1<br>NO 2<br>**(»NEXT ITEM)**	K02<br>ITEM CODE	K03 How much was purchased/paid for?<br>QUANTITY	UNIT CODES IN SECTION I	KSHS	K04 How much was acquired/obtained from other sources?<br>QUANTITY	UNIT CODES IN SECTION I	KSHS
**Medical Care**		3700						
X-rays		3709						
Lab tests		3710						
Operation/Surgery		3711						
Delivery		3712						
Room charges (health facility)		3713						
Room charges (Non Health Facility)		3714						
Ambulance charges		3715						

PROMPT FOR EACH ITEM ON THE LIST.

Over the past one month, did any member of the household acquire, purchase or consumed any [...]?	K01<br>YES 1<br>NO 2<br>**(»NEXT ITEM)**	K02<br>ITEM CODE	K03 How much was purchased/paid for?<br>QUANTITY	CODES IN SECTION I	KSHS	K04 How much was acquired/obtained from other sources?<br>QUANTITY	CODES IN SECTION I	KSHS

SECTION L: NON-FOOD EXPENDITURES – PAST ONE MONTH

PROMPT FOR EACH ITEM ON THE LIST.

Over the past one month, did any member of the household acquire or purchase(paid for) any [...]?	L01<br>YES 1<br>NO 2<br>(»NEXT ITEM)	L02<br>ITEM CODE	L03 How much was purchased/paid for?<br>QUANTITY	UNIT CODES IN SECTION I	KSHS	L04 How much was acquired/obtained from other sources?<br>QUANTITY	UNIT CODES IN SECTION I	KSHS
**Men's Clothing**		3800						
Men's Coat/Jacket		3801						
Men's Suit		3802						
Men's Trousers		3803						
Men's Shirt		3804						
Men's Underpants		3805						
Men's Sweater		3806						
Men's Vests		3807						
Men's Socks		3808						
Men's pyjamas		3809						
Ties /Scarves		3810						
**Women's Clothing**		3900						
Women's Brassiers		3901						
Women's stockings		3902						
Women's slip		3903						
Women's kitenge 1		3904						
Headsquare		3905						
Women's Dress		3906						
Women's Blouses		3907						
Women's Skirts		3908						
Women's Jacket		3909						
Women's Underwear		3910						
Women's Sweaters		3911						

PROMPT FOR EACH ITEM ON THE LIST.

Over the past one month, did any member of the household acquire or purchase(paid for) any [...]?	L01<br>YES 1<br>NO 2<br>(»NEXT ITEM)	L02<br>ITEM CODE	L03 How much was purchased/paid for?<br>QUANTITY	UNIT CODES IN SECTION I	KSHS	L04 How much was acquired/obtained from other sources?<br>QUANTITY	UNIT CODES IN SECTION I	KSHS
**Women's Clothing**		3900						
Women's Night dress		3912						
Women's Trousers		3913						
Women's suits		3914						
Ties /Scarves		3915						
**Children's Clothing**		4000						
Shirt		3901						
Trousers/Shorts		3902						
Coats/Jackes		3903						
Suit		3904						
Sweater		3905						
Underwear		3906						
Socks		3907						
vest		3908						
Girl's blouse		3909						
Girl's Skirt		3910						
Girl's Dress		3911						
Girl's Slips		3912						
Girl's Socks		3913						
Girl's Night dress/Night wear		3914						
**Infant's Clothing**		4100						
Shirt		4101						
Trousers/shorts		4102						

PROMPT FOR EACH ITEM ON THE LIST.

Over the past one month, did any member of the household acquire or purchase(paid for) any [...]?	L01<br>YES 1<br>NO 2<br>(»NEXT ITEM)	L02<br>ITEM CODE	L03 How much was purchased/paid for?<br>QUANTITY	UNIT CODES IN SECTION I	KSHS	L04 How much was acquired/obtained from other sources?<br>QUANTITY	UNIT CODES IN SECTION I	KSHS
**Infant's Clothing**		4100						
Coats/jackets		4103						
Plastic pants		4104						
Bibs		4105						
Rompers		4106						
Napkins		4107						
Sweaters		4108						
Suit		4109						
Blouse		4110						
Skirt		4111						
Dress		4112						
Socks		4113						
**Dress /clothing material**		4200						
Kanga /Kikoi		4201						
Knitting wool		4202						
Thread		4203						
Tailoring services		4204						
Needles		4205						
Buttons		4206						
Zips		4207						
Belts		4208						
Needles		4209						
Buttons		4210						
Zips		4211						
Belts		4212						

PROMPT FOR EACH ITEM ON THE LIST.

Over the past one month, did any member of the household acquire or purchase(paid for) any [...]?	L01<br>YES 1<br>NO 2<br>(»NEXT ITEM)	L02<br>ITEM CODE	L03 How much was purchased/paid for?<br>QUANTITY	UNIT CODES IN SECTION I	KSHS	L04 How much was acquired/obtained from other sources?<br>QUANTITY	UNIT CODES IN SECTION I	KSHS
**Men's Footwear**		4300						
Men's Shoes - Leather		4301						
Men's Shoes - plastic		4302						
Men's Shoes - Rubber		4303						
Men's Sports Shoes		4304						
Men's Rubber Sandals		4305						
Sandals(Akalas)		4306						
**Women's Footwear**		4400						
Women's Shoes - Leather		4401						
Women's Shoes - Plastic		4402						
Women's Shoes - Rubber		4403						
Women's Sports Shoes		4404						
Women's rubber sandals		4405						
**Boy's Footwear**		4500						
Boy's shoes - plastic		4501						
Boy's Sports Shoes		4502						
Boy's Rubber Sandals		4503						
**Girl's Footwear**		4600						
Girls' leather shoes		4601						
Sports's shoes - plastic		4602						
Girls's Sports Shoes -leather		4603						
Girls's Rubber Shoes		4604						
Repair to footwear		4605						

SECTION M: EXPENDITURES ON DURABLES

PROMPT FOR EACH ITEM ON THE LIST.

	M01	M02	M03		M04	M05	M06	M07
Over the past one year (twelve months), did any member of the household purchase or pay for any [….]	YES…1<br>NO….2<br>(»M04)	**Item code**	How much did you pay in total?		Does your household own any […..]<br>YES…….1<br>NO……..2<br>>> NEXT ITEM	How many (ITEMS) do you own?	What is the age of this (ITEM)? **If more than one item Average age**	If you wanted to sell one of this [ITEM] today, how much would you receive? **If more than one item average value**
			Number	KSHS		NUMBER	YEARS	KSHS
**Furniture**		4700						
Sofa set		4701						
Dining table		4702						
Coffee table		4703						
Dressing table		4704						
Writing/study table		4705						
Chairs		4706						
Bed		4707						
Stools wooden		4708						
Book shelves		4709						
Wardrobes		4710						
iron board		4711						
Side board		4712						
Chest of drawers		4713						
Wall unit		4714						
**Baby Goods**		4800						
Baby furniture		4801						
Baby carriages/prams		4802						
Baby cot		4803						
Walkers		4804						
Feeding bottle		4805						
Potty		4806						

	M01	M02	M03		M04	M05	M06	M07
Over the past one year (twelve months), did any member of the household purchase or pay for any [….]	NO….2<br>(»M04)	**Item code**	How much did you pay in total?		Does your household own any […..]<br>YES…….1<br>>> NEXT ITEM	How many (ITEMS) do you own?	What is the age of this (ITEM)? **If more than one item Average age**	If you wanted to sell one of this [ITEM] today, how much would you receive? **If more than one item average value**
			Number	KSHS		NUMBER	YEARS	KSHS
**Major Household Appliances**		4900						
Refrigerator		4901						
Freezers		4902						
Washing machine		4903						
Electric/gas cooker		4904						
jiko-charcoal		4905						
Micro-wave oven		4906						
Kerosene stove		4907						
Air conditioner		4908						
Electric heater		4909						
Fans		4910						
Vacuum cleaner		4911						
Electric Iron		4912						
Iron - Charcoal		4913						
Sewing machine		4914						
Electric blender		4915						
Electric toaster		4916						
Electric kettle		4917						
Coffee mills/makers		4918						
Food mixers, deep fryers		4919						
Paraffin lamps		4920						

PROMPT FOR EACH ITEM ON THE LIST.

Over the past one year (twelve months), did any member of the household purchase or pay for any [….]	M01<br>YES…1<br>NO….2<br>(»M04)	M02<br>Item code	M03<br>How much did you pay in total?		M04<br>Does your household own any […..]<br>YES…….1<br>NO……..2<br>>> NEXT ITEM	M05<br>How many (ITEMS) do you own?	M06<br>What is the age of this (ITEM)? **If more than one item Average age**	M07<br>If you wanted to sell one of this [ITEM] today, how much would you receive? **If more than one item average value**
			Number	KSHS		NUMBER	YEARS	KSHS
**Glassware, Tableware and Utensils**		5000						
Glasses		5001						
Cups and saucer		5002						
bowls		5003						
plates		5004						
spoons		5005						
knives		5006						
forks		5007						
cooking sufurias		5008						
Mwiko		5009						
frying pans		5010						
Pressure cooker		5011						
Buckets/basins		5012						
**Household Furnishings**		5100						
Carpets		5101						
Door mats		5102						
Linoleum/Synthetic carpet		5103						
Curtains and accessories		5104						
Bed covers		5105						
Bed sheets		5106						
Blankets		5107						
Pillows		5108						

Over the past one year (twelve months), did any member of the household purchase or pay for any [….]	M01<br>NO….2<br>(»M04)	M02<br>Item code	M03<br>How much did you pay in total?		M04<br>Does your household own any […..]<br>YES…….1<br>>> NEXT ITEM	M05<br>How many (ITEMS) do you own?	M06<br>What is the age of this (ITEM)? **If more than one item Average age**	M07<br>If you wanted to sell one of this [ITEM] today, how much would you receive? **If more than one item average value**
			Number	KSHS		NUMBER	YEARS	KSHS
**Household Furnishings**		5100						
Mattress		5109						
Towels		5110						
Table cloth/mats		5111						
Mosquito net		5112						
Pillow cases		5113						
**Transport and Communication**		5200						
Spark plugs/points		5201						
Clutch plate		5202						
Brake lining		5203						
Brake pads		5204						
Fuel filter		5205						
Oil filter		5206						
Other car parts specify		5207						
Car Alarm		5208						
Car Insurance		5209						
Driving lessons		5210						
International flights		5211						
Telephone installation		5212						
Cellular handset		5213						
Post office private rental box		5214						
Car		5215						

PROMPT FOR EACH ITEM ON THE LIST.

Over the past one year (twelve months), did any member of the household purchase or pay for any [….]	M01<br>YES…1<br>NO….2<br>(»M04)	M02<br>Item code	M03<br>How much did you pay in total?		M04<br>Does your household own any […..]<br>YES.......1<br>NO……..2<br>>> NEXT ITEM	M05<br>How many (ITEMS) do you own?	M06<br>What is the age of this (ITEM)? **If more than one item Average age**	M07<br>If you wanted to sell one of this [ITEM] today, how much would you receive? **If more than one item average value**
			Number	KSHS		NUMBER	YEARS	KSHS
**Transport and Communication**		5200						
Pick up		5216						
Motorcycle		5217						
Bicycle		5218						
Animal cart		5219						
Boat/canoe		5220						
Outboard engine		5221						
Computer		5222						
Satellite/decoder (cable TV) s		5223						
Radio		5224						
Television and antennae		5225						
**Recreation and Culture**		5300						
Cassette/CD/DVD -Blank		5301						
Records musical		5302						
Photography Service (Portraits, weddings,etc		5303						
V video- recorders/cameras		5304						
Radio/Cassette/CD Player		5305						
Hi -Fi Stereo		5306						
Pre-Recorded cassettes		5307						
Other musical instruments e.g. guitar		5308						
Pianos/organs		5309						
Calculators		5310						
Toys and games		5311						
Hotel Accommodation		5312						
Tour Packages		5313						

Over the past one year (twelve months), did any member of the household purchase or pay for any [….]	M01<br>NO….2<br>(»M04)	M02<br>Item code	M03<br>How much did you pay in total?		M04<br>Does your household own any […..]<br>YES.......1<br>>> NEXT ITEM	M05<br>How many (ITEMS) do you own?	M06<br>What is the age of this (ITEM)? **If more than one item Average age**	M07<br>If you wanted to sell one of this [ITEM] today, how much would you receive? **If more than one item average value**
			Number	KSHS		NUMBER	YEARS	KSHS
**Recreation and Culture**		5300						
Club membership fees		5314						
Sports/games charges		5315						
Other recreation/entertainment (specify)		5316						
**Other Non-consumption Expenditure**		5400						
Fees for Legal Services		5401						
Memberships of Professional Associations		5402						
Birth/Marriage/Death Certificates		5403						
Coffin, Funeral Urns, and Tombstones		5404						
Road Licenses		5405						
Newspaper Notices/Advertisements		5406						
Service of Marriage Counselors		5407						
Fines		5408						
Money Lost or Stolen (unrecovered)		5409						
Life insurance		5410						
House insurance		5411						
Education insurance		5412						
Other insurance		5413						
Non Agricultural land		5414						
Borehole construction/repair		5415						
Water purification systems		5416						
Fixed line telephone costs		5417						
Sewage collection		5418						
Toilet emptying services		5419						

SECTION N: AGRICULTURE HOLDING

[ASK OF ALL THOSE CONCERNED WITH FARMING IN THE HOUSEHOLD]. THE TIME REFERENCE IS THE LAST 12 MONTHS.
LIST IN N03 ALL PARCELS BEFORE COLLECTING DETAILS ON EACH.

N01: Did any member of the household engage in farming in the last 12 months, whether self-employed or as a tenant? [ ] YES 1 NO 2 (»SECTION P)

N02	N03	N04	N05	N06	N07	N08					N09	N10	N11	N12	N13	N14
PARCEL ID	Please tell me about each parcel of land that a HH member farmed during the last 12 months within this district.<br>WRITE A NAME TO IDENTIFY PARCEL	Who in the household makes the decisions on input use and cropping activities on this parcel?<br>IF PRESENT, ASK FOLLOWING QUESTIONS TO THIS PERSON.<br>ID CODE<br>NON MEMBER….98	What is the size of the parcel? LAND AREA IN ACRES (one decimal place)<br>DK 98<br>ACRES	What is the general texture of the soil on this parcel:<br>LOAMY 1<br>SANDY 2<br>BETWEEN SANDY & CLAY 3<br>CLAY 4<br>OTHER 5	What is the slope of this parcel:<br>FLAT 1<br>SLIGHT SLOPE 2<br>MODERATE SLOPE 3<br>STEEP/ HILLY 4<br>OTHER 5	What crops were grown on this parcel in the last 12 months? LIST MOST IMPORTANT FIRST. SEE CODES IN MANUAL<br>1st	2nd	3rd	4th	5th	Does HH own this parcel?<br>YES 1<br>NO 2 (»N11)	Does HH have a title?<br>YES 1 (»N12)<br>NO 2 (»N12)	How does HH operate this parcel (status of operations)?<br>RENTED/ LEASED 1<br>FREE -FAMILY 2<br>FREE-OTHERS 3<br>COMMUNAL 4<br>OTHER (SPEC.) 5	Do people buy and sell farmland in this area?<br>YES 1<br>NO 2 (»N14)	If HH were to sell/buy this parcel today, how much could it fetch?<br>KSHS	How much did you pay in total for your rented/leased parcel in last 12 months?<br>ESTIMATE VALUE OF ANY IN-KIND PAYMENTS.<br>answer if N11 = 1<br>KSHS
2																
3																
4																
5																
6																
7																
8																
9																
10																
11																
12																

N02<br>PARCEL ID	N15<br>How many months in the last 12 months did HH practice irrigation on this parcel of land?<br>IF 0 MONTHS<br>(»N20)	N16<br>What type of irrigation is practised on this parcel?<br>WATERING CAN 1<br>HOSEPIPE 2<br>SPRINKLER 3<br>CANALS 4<br>FURROW 5<br>DRIP 6<br>OTHER (SPECIF' 7	N17<br>How does HH get the water for irrigation on this parcel?<br>DIVERT STREAM 1<br>BUCKET FROM WELL 2<br>HANDPUMP FROM WEL 3<br>TREADLE PUMP 4<br>MOTORPUMP 5<br>GRAVITY-FED PIPELINE 6<br>OTHER (SPECIFY) 7	N18<br>How much did HH spend on irrigation water on this parcel?<br>KSHS	N19<br>In how many months does HH have sufficient water for irrigation of this parcel?<br>MONTHS	N20<br>Did HH apply any inorganic fertilizer to this parcel in the last 12 months?<br>YES 1<br>NO 2<br>(»N24)	N21<br>How much inorganic fertilizer (Kgs) did the HH apply in the last 12 months?<br>KGS	N22<br>How much did HH pay for the inorganic fertilizer used on this parcel in the last 12 months?<br>ESTIMATE VALUE OF ANY IN-KIND PAYMENTS.<br>KSHS	N23<br>Where did HH acquire the fertilizer?<br>LIST UP TO 2.<br>MERCHANT 01<br>RELATIVE 02<br>NEIGHBOR/FRIEND 03<br>LOCAL MARKET 04<br>COOP/ASSOC 05<br>COMPANY 06<br>NGO/CBO 07<br>GOK /GOK AGENCI 08<br>OTHER (SPECIFY) 09<br>1st	2nd	N24<br>Did HH apply any organic fertilizer to this parcel in the last 12 months?<br>ANIMAL MANURE, COMPOST, GREEN MANURE<br>YES 1<br>NO 2<br>(»N26)	N25<br>How much did HH pay for the organic fertilizer used on this parcel in the last 12 months?<br>ESTIMATE VALUE OF ANY IN-KIND PAYMENTS.<br>KSHS
2												
3												
4												
5												
6												
7												
8												
9												
10												
11												
12												

N02	N26<br>How much did the HH spend (Kshs.) on the following agricultural inputs in the last 12 month?										N27<br>What was the HH expenditure in Kshs. on the following items in the last 12 months		
PARCEL ID	PESTICIDES	FUEL	LUBRICANTS	ELECTRICITY	MACHINE/ EQUIP REPAIRS	TRACTOR/ OXEN PLOUGH	PURCHASE OF SMALL FARM IMPLIMENTS	FARM REPAIR	LABOUR COSTS	OTHERS	TOTAL<br>Total	LAND RECLAMATION, CLEARANCE ETC.	ESTABLISHMENT OF LONG TERM CROPS E.G. COFFEE, COTTON ETC	PRCHASE OF MECHANICAL EQUIPMENT E.G. TRACTOR
2														
3														
4														
5														
6														
7														
8														
9														
10														
11														
12														

SECTION O: AGRICULTURE OUTPUT

[ASK OF ALL THOSE CONCERNED WITH FARMING IN THE HOUSEHOLD]. THE TIME REFERENCE IS THE LAST 12 MONTHS.
LIST ALL CROPS BEFORE COLLECTING DETAILS ON EACH.

O01	O02		O03	O04	O05	O06		O07	O08	O09	O10	O11	O12		O13		O14		O15	
	Please tell me all the crops that members of the household farmed in the past 12 months, including **cash crops, tree crops and food crops**.<br><br>CHECK THAT ALL CROPS LISTED IN SECTION N ARE LISTED		On which parcels did HH farm [NAME CROP in O02] ?<br><br>RECORD PARCEL IDS LISTED IN **SECTION N**	Total crop area in **ACRES** (one decimal place)	Did HH use any purchased seeds for this crop in the last 12 months?<br><br>YES 1<br>NO 2<br>(»O09)	Where did HH acquire the seeds?<br>LIST UP TO TWO.<br><br>MERCHANT 01<br>RELATIVE 02<br>NEIGHBOR 03<br>LOCAL MARKET 04<br>COOP/ASSOC. 05<br>PRIVATE COMPANY 06<br>NGO/CBO 07<br>GOK AGENCIES 08<br>OTHER (SPECIFY) 09		What quantity of seeds did HH obtain ?	How much did HH pay for the purchased seeds used for this crop?<br><br>ESTIMATE VALUE OF ANY IN-KIND PAYMENTS.	Did HH use any purchased seedlings for this crop in the last 12 months?<br><br>YES 1<br>NO 2<br>(»O13)	What number of seedlings did HH obtain?	How much did HH pay for the purchased seedlings used for this crop?<br><br>ESTIMATE VALUE OF ANY IN-KIND PAYMENTS.	Where did HH acquire the seedlings?<br>LIST UP TO TWO.<br><br>MERCHANT 01<br>RELATIVE 02<br>NEIGHBOR/FRIEND 03<br>LOCAL MARKET 04<br>COOP/ASSOC. 05<br>PRIVATE COMPANY 06<br>NGO/CBO 07<br>GOK AGENCIES 08<br>OTHER (SPECIFY) 09		How much of the [...] did the HH harvest in the last 12 months from all these parcels in which [...] was planted?<br><br>IF NONE, ENTER ZERO<br>>>Next crop		How much of the [...] harvested in the last 12 months was consumed?<br><br>IF NONE, ENTER ZERO		How much of the [...] harvested in the last 12 months was sold?<br><br>IF NONE, ENTER ZERO.<br>**>> O19**	
	CROP NAME	CROP ID	PARCEL ID	ACRES		1st	2nd	Kgs	KSHS		number	KSHS	1st	2nd	QUANTITY	UNIT CODES BELOW	QUANTITY	UNIT CODES BELOW	QUANTITY	UNIT CODES BELOW
1																				
2																				
3																				
4																				
5																				
6																				
7																				
8																				
9																				
10																				
11																				
12																				

O13 - O21

UNIT	
KILOGRAMME. . 1	Tonnes. . .4
50 KG. BAG. . 2	Number . . 5
90 KG. BAG. . 3	

O18

MERCHANT. . . 1	LOCAL MARKET. . 4	GOK AGENCIES. . 7
RELATIVE. . . 2	COOP/ASSOC. . . 5	AUCTION FLOOR 8
NEIGHBOR. . . 3	PRIVATE COMPANY 6	OTHER (SPECIFY).9

O01	O16<br>What was the total value earned from the [...] sale?	O17<br>What was the average price paid for the crop during the last 12 months?	O18<br>To whom was [...] sold to? LIST UP TO 2 BUYERS.		O19<br>After harvest, how much [...] was used to pay labourers or make other payments?<br>IF NONE, ENTER ZERO.		O20<br>How much of the [...] harvested in the last 12 months is still being stored by household?<br>IF NONE, ENTER ZERO.		O21<br>How much of the [...] harvested in the last 12 months was used as seeds by household?<br>IF NONE, ENTER ZERO.		O22<br>How much of the [...] harvested in the last 12 months was given out as gifts by the household?<br>IF NONE, ENTER ZERO.		O23<br>How much of the [...] harvested in the last 12 months was lost / wasted by household?<br>IF NONE, ENTER ZERO and >>O25.		O24<br>What was the cause of the loss or wastage?<br>LIST TWO MAIN REASONS<br>WEEVILS 1<br>MICE/RAT 2<br>FLOOD 3<br>WILD ANIMALS 4<br>THEFT 5<br>OTHER (SPECIFY) 6		O25<br>For this crop, how many days of labour did HH hire? Include all tasks - clearing, ridging, planting, weeding, harvest.<br>IF NONE, ENTER ZERO and >>next CROP	O26<br>How much did HH pay for the total amount of labour used for this crop in the last 12 months?<br>'ESTIMATE VALUE OF ANY IN-KIND PAYMENTS.
	KSHS	KSHS/Unit	CODES BELOW 1	2	QTY	UNIT CODES BELOW	QTY	UNIT CODES BELOW	QTY	UNIT CODES BELOW	QTY	UNIT CODES BELOW	QTY	UNIT CODES BELOW	REASON 1	REASON 2	MAN-DAYS OF LABOUR	KSHS
1																		
2																		
3																		
4																		
5																		
6																		
7																		
8																		
9																		
10																		
11																		
12																		

SECTION P: LIVESTOCK

[ASK OF THOSE CONCERNED WITH ANIMAL HUSBANDRY IN THE HOUSEHOLD]. THE TIME REFERENCE IS THE LAST TWELVE MONTHS.

P01: Has any member of your household raised or owned livestock, poultry, fish, bees etc during the past 12 months? [ ]

Yes 1
No 2 (»SECTION Q)

ID CODE	P02 Please give me the types of all animals that any member of the household raised in the last twelve months. *See the manual for livestock codes*		P03 How many […] does household own at present?	P04 If household sold one of those […] today, how much money could household get for it? *(AVG. PRICE)*	P05 How many […] did household sell during the last twelve months? *IF NONE, ENTER ZERO* IF ZERO, »P07	P06 How much did household receive for the sale of all these […] during the last 12 months? *INCLUDE VALUE OF IN-KIND PAYMENTS*	P07 How many [. . .] did household consume during the last 12 months? *IF NONE, ENTER ZERO*	P08 How many […] died during the last 12 months? *IF NONE, ENTER ZERO*	P09 How many of your […] were lost or were stolen during the last 12 months? *IF NONE, ENTER ZERO*	P10 How many of your […] were given away during the last 12 months? *IF NONE, ENTER ZERO*
	ANIMAL	ANIMAL CODE	No. OF ANIMALS	KSHS	No. OF ANIMALS	KSHS	No. OF ANIMALS	No. OF ANIMALS	No. OF ANIMALS	No. OF ANIMALS
1										
2										
3										
4										
5										
6										
7										
8										
9										
10										
11										
12										

ID CODE	P11	P12 Did household purchase any (….) in the last 12 months	P13 How many […] did household purchase during the last 12 months?	P14 How much did household pay in total for these […] during the last 12 months? INCLUDE VALUE OF IN-KIND PAYMENTS	P15 How many […] were born during the last 12 months? IF NONE, ENTER ZERO	P16 How many […] were received by your household during the last 12 months? IF NONE, ENTER ZERO	P17 For all your (…), how many days of labour did HH hire in the last 12 months? Include all tasks - (herding, feeding, milking etc) IF NONE, ENTER ZERO and »P19	P18 How much did HH pay for the total amount of labour used on this (…) in the last 12 months? ESTIMATE VALUE OF ANY IN-KIND PAYMENTS.	P19 How much did the HH spend on the following livestock inputs in the last 12 month?								
		YES 1<br>NO 2							Drugs and medicines	Vaccines	Water	Livestock chemicals(dipping and spraying	Livestock manufactured feeds	Livestock fodder	Artificial Insemination	Livestock Insurance	
	ANIMAL CODE	(»P15)	No. OF ANIMALS	KSHS	No. OF ANIMALS	No. OF ANIMALS	PERSON-DAYS OF LABOUR	KSHS	KSHS	KSHS	KSHS	KSHS	KSHS	KSHS	KSHS	KSHS	ANIMAL CODE
1																	
2																	
3																	
4																	
5																	
6																	
7																	
8																	
9																	
10																	
11																	
12																	

I D CODE	P19 How much did the HH spend on the following livestock inputs in the last 12 month?								P20 Who is the main provider of Artificial Insemination? FOR CATTLE ONLY	P21 Who is the main provider of veterinary (animal treatment) services?	P22 What was the household expenditure in KSHS on the following items in the last 12 months?	
	fuels<br>KSHS	lubricants<br>KSHS	electricity<br>KSHS	machinery/equipment repairs<br>KSHS	purchase of small farm implements<br>KSHS	Farm repair<br>KSHS	Other<br>KSHS	Total<br>KSHS	Government 1<br>Private Vet 2<br>Self 3<br>None 4<br>Others specify 5	Government 1<br>Private Vet 2<br>Self 3<br>None 4<br>Others specify 5	Cattle sheds, paddocks and other livestock constructions<br>Kshs	Purchase of mechanical equipments eg. Tractor,Ox Ploughs etc.<br>Kshs
1												
2												
3												
4												
5												
6												
7												
8												
9												
10												
11												
12												

ID CODE	P23 What quantity of (PRODUCE) did you get in the last one month? SEEE CODES ON THE RIGHT SIDE			P24 Of what was produced how much was sold? IF NONE, ENTER ZERO and **>>P26** Codes below SEEE CODES ON THE RIGHT SIDE		P25 To whom was the produce sold? See codes below	P26 What quantity of produce was given out?	
		Quantity	unit	Quantity	Value(KSHS)		Quantity	Unit
1	Milk							
2	Eggs							
3	Honey							
4	Wool/fur							
5	Hides/skins							
6	Beef							
7	Pork							
8	Mutton/Goat meat							
9	Fish							
10	Chicken meat							
11	Other							
12								

P23,P24,P26

Kilogramme	1
Litre	2
Number	3
Trays	4
5-litre jerry can	5

P25

co-operative. ...	1
KCC 2	2
Factory/Trader..	3
KFA 4	4
Local Mkt	5
Roadside	6
On Farm	7
Company	8
Others	9

SECTION Q: HOUSEHOLD ENTERPRISES

[ASK OF HOUSEHOLD HEAD OR MOST KNOWLEDGEABLE RESPONDENT]

Q01. Over the past month, has anyone in your household operated any **non-agricultural income-generating enterprise** which produces goods or services or has anyone in your household owned a shop or operated a trading business?<br>(Enterprises include posho mill , jua kali business, water kiosks/vendor, solid waste collection, etc. (informal enterprises)	☐ YES. 1<br>NO . 2<br>(»NEXT SECTION)

Q02	Q03		Q04	Q05		Q06	Q07		Q08	Q09	Q10		Q11	Q12		Q13	
	What income-generating activities did individuals in the household operate over the past month?<br>**COLLECT INFORMATION ON ALL income-generating activities HERE BEFORE GOING ON TO COLLECT DETAILS ON EACH.**		Type of income generating activity<br>SINGLE Product/Service 1<br>MIXED (2 products/service) 2<br>MIXED (more than 2 products/service) 3	Who in the household owns this income-generating activity?<br>**CAN LIST UP TO TWO OWNERS. ID CODES MUST BE FROM HH ROSTER**		How many individuals outside of the household are co-owners of this income generating activity?<br>**IF NONE, WRITE 0.**	Who in the household manages this income generating activity or is most familiar with it?<br>**IF CO-MANAGERS, LIST BOTH. IF PRESENT, ASK FOLLOWING QUESTIONS TO MANAGER(S).**		How many months during the last 12 months did householdoperate this income generating activity?	Where does household operate the income generating activity?<br>HOME, INSIDE RESIDENCE 01<br>HOME OUTSIDE RESIDENCE 02<br>JUA KALI Sheds 03<br>LOCAL MARKET 04<br>COMMERCIAL AREA SHOP 05<br>ROADSIDE 06<br>OTHER FIXED PLACE 07<br>MOBILE 08<br>KIOSKS 09<br>OTHER (Specify) 10	How long, has this income generating activity been in existence?		Is this income-generating activity officially registered with the Registrar of Companies?<br>YES 1<br>NO 2<br>DK 3	How many household members are engaged in this income-generating activity?<br>**IF NONE, ENTER ZERO**		How many employees are there who are not household members in the last one month?<br>IF NONE, ENTER ZERO	
	WRITTEN DESCRIPTION	4 DIGIT CODE BY SUP		OWNER 1 ID CODE	OWNER 2 ID CODE	NUMBER	MAN. 1 ID CODE	MAN. 2 ID CODE	NUMBER OF MONTHS		YEARS	MONTHS		MALES	FEMALE	MALES	FEMALE
1																	
2																	
3																	
4																	
5																	
6																	
7																	
8																	
9																	
10																	
11																	
12																	

Q02	Q14<br>When the income-generating activity is running, how many hours per week does each employee work on average?<br>HOURS PER WEEK	Q15<br>What was the main source of start-up capital for this income-generating activity?<br>CAN LIST UP TO THREE.<br>LOAN FROM FAMILY/FRIENDS 01<br>GIFT FROM FAMILY/FRIENDS 02<br>SALE OF ASSETS OWNED 03<br>PROCEEDS FROM ANOTHER BUSINESS 04<br>OWN SAVINGS 05<br>LOAN FROM SACCOS 06<br>NON-AGRICULTURAL CREDIT, BANK OR OTHER INSTITUTION 07<br>LOAN FROM MONEY LENDER 08<br>INHERITED 09<br>OTHER (SPECIFY) 10<br>NONE 11<br>1st	2nd	3rd	Q16<br>What were the total sales for the income-generating<br>KSHS	Q17<br>What were the business costs last month in the following categories?<br>**IF NONE, ENTER ZERO**<br>Rent<br>KSHS	Salaries, wages<br>KSHS	Licences and Taxes<br>KSHS	Electricity and water<br>KSHS	Insurance<br>KSHS	purchase of business wares and Goods for resale<br>KSHS	Transport<br>KSHS	purchase of inputs and other raw materials for producing final goods or for providing services<br>KSHS	Other<br>KSHS	TOTAL COSTS<br>KSHS	Q18<br>To whom do you sell your products or services?<br>LIST UP TO 2 BUYERS.<br>FINAL CONSUMERS 01<br>SMALL BUSINESS 02<br>LARGE ESTABLISHED BUSINESS INSTITUTIONS 04<br>EXPORT 05<br>MANUFACTURERS 06<br>GOVERNMENT 07<br>OTHER (SPECIFY) 08<br>1st	2nd	Q19<br>Over the past six months, did you earn a profit, make a loss, or just break even?<br>EARNED A PROFIT 1<br>LOSS 2<br>BROKE EVEN 3<br>(**»NEXT Economic activity**)	Q20<br>What was the amount you earned or lost from this income generating activity over the past six month?<br>**IF A LOSS (COSTS GREATER THAN SALES), PUT FIGURE IN PARENTHESES.**<br>KSHS
1																			
2																			
3																			
4																			
5																			
6																			
7																			
8																			
9																			
10																			
11																			
12																			

SECTION R: TRANSFERS

[ASK OF HOUSEHOLD HEAD OR MOST KNOWLEDGEABLE RESPONDENT]

R01: Has the household received or given out any transfers (cash or in-kind) (include CEREMONIAL EXPENDITURES) during past 12 months? [ ]

YES. 1
NO . 2
(»R10)

R02	R03					R04					R05					R06	R07	R08	R09
Over the past 12 months, did household <u>receive</u> any gifts (in cash or in-kind) from any source (Individuals / family/institutions) outside your household?	What was the total value of all <u>cash</u> received as a gift from (…..) in the last 12 months? Kshs					What was the total value of all <u>food</u> received as a gift from (….) in the last 12 months? Kshs					What was the total value of <u>all other in-kind gifts</u> received from (…) in the last 12 months? Kshs					Over the past 12 months, did household <u>give out</u> any gifts (in cash or in-kind) to any individuals/institutions?	What was the total value of all <u>cash</u> given as a gift to individuals/institution in the last 12 months?	What was the total value of all <u>food</u> given as a gift to individuals/institution in the last 12 months?	What was the total value of <u>all other in-kind gifts</u> to individuals/institutions in the last 12 months?
YES 1<br>NO 2<br>(»R06)	Individual	Non profit Institution	Government	Corporate Sector	Outside Kenya	Individual	Non profit Institution	Government	Corporate Sector	Outside Kenya	Individual	Non profit Institution	Government	Corporate Sector	Outside Kenya	YES 1<br>NO 2<br>(»R10)	KSHS	KSHS	KSHS

R10	R11			
Which individuals in the household were members of a self-help group in the past 12 months?	Type of self-help group member belongs to<br>WOMEN'S/MEN'S GROUP 1<br>MERRY-GO-ROUND 2<br>YOUTH-RELATED 3<br>RELIGIOUS-RELATED 4<br>FAMILY-RELATED 5<br>SOCIAL WELFARE GROUPS 6<br>OTHER (SPECIFY) 7			
**ID CODE**	TYPE 1	TYPE 2	TYPE 3	TYPE 4

SECTION S: OTHER INCOME

[ASK OF HOUSEHOLD HEAD OR MOST KNOWLEDGEABLE RESPONDENT]

S01: Has any member of your household received any other income (**NOT MENTIONED ELSEWHERE**) in the past 12 months? [ ] YES. 1 NO . 2 (»NEXT SECTION)

S02	S03	S04	S05	S06	S07					S08	S09		S10	
Do any members of your household receive any regular income from savings, interest or other investment income?	How much does your household receive in savings, interest or other investment income?	Do any members of your household receive any regular income from a pension?	How much did your household receive in pension income in the last month?	Do any members of your household receive any regular income from rental of property	How much does your household usually receive in rental income per month?					Do any members of your household receive any regular income of any other type?	What type of income? (Describe)		How much does your household usually receive from this other income(s) per month in KSHS?	
YES 1<br>NO 2<br>(»S04)	KSHS	YES 1<br>NO 2<br>(»S06)	KSHS	YES 1<br>NO 2<br>(»S08)	RESIDENTIAL<br>KSHS	COMMERCIAL<br>KSHS	LAND<br>KSHS	SUB SOIL ASSETS<br>KSHS	OTHER PROPERTY (Specify)<br>KSHS	YES…….1<br>NO …….2<br>(» S11)	Type 1	Type 2	Type 1	Type 2

S11	S12		S13
Did any member of your HH receive any other sort of income (Non Regular) in the last 12 months	What type of income (Describe)		How much in total did the HH receive in form of this income(s) (Non Regular) in the last 12 months?
YES 1<br>NO 2<br>(»NEXT SECTION)	Type 1	Type 2	KSHS

SECTION T: RECENT SHOCKS TO HOUSEHOLD WELFARE

[ASK OF HOUSEHOLD HEAD OR MOST KNOWLEDGEABLE RESPONDENT]

T01<br><br>CODE	T02<br><br>Over the past **five years**, was your household severely affected negatively by any of the following events?<br><br>**GO THROUGH ENTIRE LIST BEFORE PROCEEDING TO T03.**	YES 1<br>NO 2<br>(»NEXT ITEM)	T03<br><br>Rank the three most significant shocks you experienced<br><br>Most severe 1<br>Second most severe 2<br>Third most severe 3	**THE QUEST-IONS TO THE RIGHT SHOULD ONLY BE ASKED CON-CERNING THE THREE MOST SEVERE SHOCKS, AS NOTED IN T03.**<br><br>**LEAVE ALL OTHER ROWS BLANK.**	T04<br><br>What was the estimated value lost due to this shock?<br><br>KSHS	T05<br><br>Did [**THIS SHOCK**] cause a reduction in household income and/or assets?<br><br>INCOME LOSS 1<br>ASSET LOSS 2<br>LOSS OF BOTH 3<br>NEITHER 4	T06<br><br>[**THIS SHOCK**] affected:<br>[READ]<br><br>OWN HH ONLY 1<br>SOME OTHER HH TOO 2<br>MOST HH IN COMMUNITY 3<br>ALL HH IN COMMUNITY 4	T07<br><br>How long ago did [**THIS SHOCK**] occur?<br><br>YEARS	 <br>MONTHS	T08<br><br>What did you do in response to [**THIS SHOCK**] to try to cope / regain your former welfare level?<br><br>[**LIST UP TO 3 BY ORDER OF IMPORTANCE, CODES AT RIGHT.**]<br><br>1ST	 <br>2ND	 <br>3RD
101	Drought or Floods											
102	Crop disease or crop pests											
103	Livestock died or were stolen											
104	Household business failure, non-agricultural											
105	Loss of salaried employment or non-payment of salary											
106	End of regular assistance, aid, or remittances from outside HH											
107	Large fall in sale prices for crops											
108	Large rise in price of food											
109	Large rise in agricultural input prices											
110	Severe water shortage											
111	Chronic/severe illness or accident of household member											
112	Birth in the household											
113	Death of HH head											
114	Death of working member of household											
115	Death of other family member											
116	Break-up of the household											
117	Jailed											
118	Fire											
119	Carjacking/Robbery/burglary/assault											
120	Dwelling damaged, destroyed											
121	HIV/AIDS											
122	Other 1 _______________											
123	Other 2 _______________											

spent cash saving	01
Sent children to live with relatives	02
Sold assets (tools, furniture etc)	03
Sold farm land	04
Rented out farm land	05
Sold animals	06
Sold more crops	07
Worked more, worked longer hours	08
Other household members who weren't working went to work	09
Started a new business	10
Removed children from school to work	11
Went elsewhere to find work for more than a month	12
Borrowed money from relatives	13
Borrowed money from money lender	14
Borrowed money from institutions (banks etc)	15
Received help from religious institutions	16
Received help from local NGO	17
Received help from international NGO	18
Received help from Government	19
Received help from family/friends	20
Reduced food consumption	21
Consumed lower cost, but less prefered foods	22
Reduced non food expenditures	23
Spiritual help- prayers, sacrifices, consulted diviner etc	24
Others specify	25

SECTION U: CREDIT

ASK OF HOUSEHOLD HEAD AND PERSONS RESPONSIBLE FOR LOANS LISTED.

U01	Over the past **12 months**, did you or anyone else in this household borrow from someone outside the household or from an institution receiving either cash, goods or services?	☐	YES. 1<br>NO . 2 >>U12

U02	U03	U04	U05	U06	U07	U08		U09	U10	U11
	What are the names of the persons or institutions from whom you or anyone else in your household borrowed over the past 12 months?<br><br>LIST ALL NAMES BEFORE GOING TO THE NEXT QUESTION.	CODE SOURCE OF LOAN<br><br>USE CODES AT LOWER RIGHT	Which house-hold member	What was main reason for obtaining loan?<br>Was it: [READ]<br><br>Subsistence needs 01<br>Medical cost 02<br>School fees 03<br>Ceremony/Wedding 04<br>Purchase land 05<br>Purchase agricultural inputs 06<br>Other business inputs 07<br>purchase agricultural Machinery 08<br>Purchase/Constructioion of dwelling 09<br>Other (Specify) 10	How much was borrowed?<br><br>ENTER THE VALUE OF ITEMS ACQUIRED ON CREDIT	When did you get the loan?<br><br>JAN 01 JUL 07<br>FEB 02 AUG 08<br>MAR 03 SEP 09<br>APR 04 OCT 10<br>MAY 05 NOV 11<br>JUN 06 DEC 12		Rate of interest per annum	Repayment period of loan in months	How much is outstanding (awaiting to be repaid)<br><br>(GO TO NEXT LOAN. WHEN ALL LOANS, DONE »U12)
			ID CODE		KSHS	CALENDAR MONTH	CALENDAR YEAR			KSHS
1										
2										
3										
4										
5										
6										
7										
8										

U12	U13		U14	
During the last **12 months**, did you try to borrow from someone outside the household or from an institution and were turned down?<br><br>YES 1<br>NO 2<br>(>> U14)<br>STILL AWAITING WORD ON LOAN 3	Who turned you down?<br>LIST UP TO 2.<br><br>USE CODE BELOW.		Why did you not attempt to borrow in the last 12 months?<br>[WRITE UP TO TWO ANSWERS IN ORDER OF IMPORTANCE.]<br><br>No need 01<br>Believed would be refused 02<br>Too expensive 03<br>Too much trouble for what it is worth 04<br>Inadequate collateral 05<br>Do not like be in debt 06<br>Do not know any lender 07<br>Others specify 08	
IF =3 END INTERVIE	1ST	2ND	1ST	2ND

CODES FOR U04 & U13

COMMERCIAL BANKS	01	GROCERY/LOCAL MERCHANT	08
MICRO-FINANCE INST.	02	MONEY LENDER (Shylock)	09
BUILDING SOC./MORTGAGE	03	EMPLOYER	10
INSURANCE COMPANIES	04	RELIGIOUS INST.	11
SACCOS	05	NGO	12
OTHER FINANCIAL INST.	06	SELF-HELP GROUPS	13
NEIGHBOURS / FRIENDS	07	OTHER (Specify)	14
